# Efficient carbon dioxide hydrogenation to formic acid with buffering ionic liquids

**DOI:** 10.1038/s41467-020-20291-0

**Published:** 2021-01-11

**Authors:** Andreas Weilhard, Stephen P. Argent, Victor Sans

**Affiliations:** 1 Faculty of Engineering, University of Nottingham, Nottingham, NG7 2RD UK; 2 School of Chemistry, University of Nottingham, Nottingham, NG7 2RD UK; 3 Institute of Advanced Materials (INAM), Universitat Jaume I, 12071 Castellon, Spain

**Keywords:** Catalyst synthesis, Homogeneous catalysis, Ionic liquids

## Abstract

The efficient transformation of CO_2_ into chemicals and fuels is a key challenge for the decarbonisation of the synthetic production chain. Formic acid (FA) represents the first product of CO_2_ hydrogenation and can be a precursor of higher added value products or employed as a hydrogen storage vector. Bases are typically required to overcome thermodynamic barriers in the synthesis of FA, generating waste and requiring post-processing of the formate salts. The employment of buffers can overcome these limitations, but their catalytic performance has so far been modest. Here, we present a methodology utilising IL as buffers to catalytically transform CO_2_ into FA with very high efficiency and comparable performance to the base-assisted systems. The combination of multifunctional basic ionic liquids and catalyst design enables the synthesis of FA with very high catalytic efficiency in TONs of >8*10^5^ and TOFs > 2.1*10^4^ h^−1^.

## Abstract (web summary)

Basic ionic liquids provide a buffering effect that enables the efficient synthesis of free formic acid from CO_2_ hydrogenation. Here, a highly efficient catalytic system that transforms CO_2_ to formic acid without the need of strong bases is demonstrated, avoiding the formation of formate salts.

## Introduction

Formic acid (FA) is an important basic chemical, is currently being discussed as a promising candidate for hydrogen storage and can potentially be upgraded to higher added value CO_2_ synthetic products^1–3^. Unfortunately, to date its synthesis is based on the indirect water carbonylation, using CO generated from fossil fuels^4^. Without a doubt a system using the greenhouse gas CO_2_ as a carbon source would be environmentally preferential, if the hydrogen used for these transformation is generated from renewable resources. However, the direct hydrogenation of CO_2_ is thermodynamically unfavourable^5^. Bases are commonly added to reaction mixture to shift the thermodynamic equilibrium by the consecutive consumption of FA in an acid–base reaction to the product side^5–7^.

However, an important limitation of these approaches is the formation of formate adducts and salts, which would need to be tediously purified in the consecutive steps with acids, which adds to the cost and the amount of waste generated^6,8^. Therefore, the direct hydrogenation of CO_2_ would be preferential. Unfortunately, the hydrogenation is in gas phase thermodynamically unfeasible^5^. Alternatively, the reaction can be undertaken in the absence of bases utilising basic properties of solvents applied during the hydrogenation^9–15^. However, in the absence of strong bases the reaction is thermodynamically and kinetically difficult to operate^5^. As a consequence, considerably fewer systems have been reported for the hydrogenation of CO_2_ to FA under base-free conditions. Furthermore, these systems typically operate under very high pressures^9^ and the catalytic activity and concentration of FA achieved are significantly lower than under basic conditions^10–15^.

We have recently demonstrated that basic ionic liquids can effectively buffer the reaction, therefore acting as very mild bases that shift the thermodynamic equilibrium to the product side, whilst stabilising the catalytically active species at low partial pressure of H_2_ and CO_2_^16^. Furthermore, we have demonstrated that buffering ILs can be immobilised onto supported phases, thus facilitating the separation of the FA at the end of the process following a supported ionic liquid approach^17–20^. In our previous studies we have established the thermodynamic parameters for this transformation employing basic ILs, and therefore we can understand the maximum concentration of FA achievable as a function of the reaction conditions^16^. Furthermore, we previously studied the effect of ligand modifications onto the hydrogenation of CO_2_ in the presence of ILs. Key parameters effecting the catalyst activity and stability were associated with the electron density at the metal centre and the resistance of the catalyst towards protonation^21^.

Here, we present a catalytic system for the hydrogenation of CO_2_ under buffering conditions specifically designed to work under a wide range of temperatures. This enables optimising the catalyst performance by pushing the conditions to the thermodynamic limitations imposed by the reaction and consequently balancing kinetic and thermodynamic performance and achieving high catalytic efficiency. The robustness of the catalyst enables the addition of lewis acids, which increases the performance of the catalyst in terms of activity and stability. The results obtained here are comparable to those reported with systems containing stronger bases and represents an important leap forward towards sustainable systems to transform CO_2_.

## Results and discussion

### Synthesis of catalyst

**Fig. 1:**
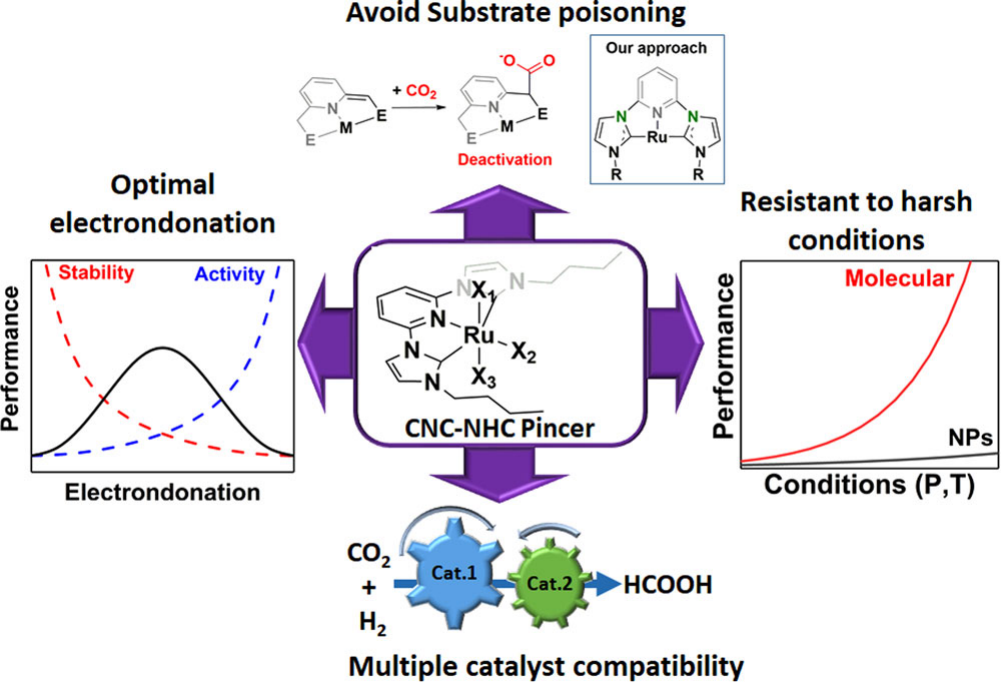
Catalyst design strategy. The molecular catalyst (centre) should be resistant to deactivation by the substrates (top), tune the electrondonation of the ligand to the metal centre to balance stability and activity (left), resist the increasingly acidic media and strong reductive conditions (right) and be compatible with other catalysts (bottom).

The design of the catalysts plays a key role in the development of efficient catalytic systems. Very recently, it has been demonstrated that basic ionic liquids provide a buffering environment that enables the efficient synthesis of FA, but with much lower enthalpy than when a base (leading to a salt formation) is employed^16,22^. Under these conditions, optimal electrondonation properties of the ligand are key to balance reactivity and the resistance to deactivation via protonation of the catalyst (Fig. 1). Furthermore, complexes with high chemical and thermal stability lead to catalysts with large operational windows and an increased compatibility with other components, such as co-catalysts. NHC-based pincer architectures fulfil all these criteria, hence being ideal candidates for this transformation^23^. Furthermore, pincer ligand architecture have already been demonstrated to be active in the hydrogenation of CO_2_. However, pincers normally exhibit a co-operative mechanism in the activation of hydrogen^24,25^, which leads to the deactivation of the catalyst (Fig. 1, substrate poisoning)^26^. Consequently, a suitable catalyst should prevent the coordination of substrates in undesired positions. In this study, this was achieved by a direct connection of the aromatic pyridine with the imidazole side arms.

**Fig. 2:**
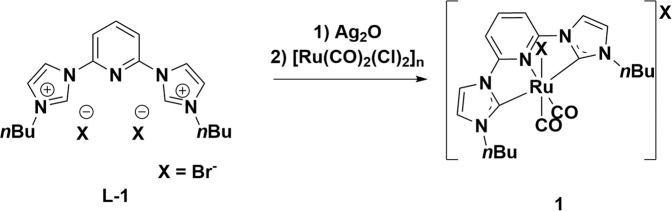
Synthetic strategy for the formation of 1. The ligand **L-1** was sequentially reacted with Ag_2_O, followed by transmetalation with [Ru(CO)_2_(Cl)_2_]_*n*_. to yield complex **1**.

The silver transmetalation route employing [Ru(CO)_2_(Cl)_2_]_*n*_ as the Ru source, and **L-1** as the ligand was chosen to synthesise the desired Ru–CNC complex (Fig. 2). The reaction was followed by the disappearing signal for the C_2_–H in the ^1^H-NMR spectra and the appearance of a new peak at 181 ppm in the ^13^C spectra (see Supplementary Figs. 1 and 2). In the IR spectra two significant bands at 1993 and 2051 cm^−1^ (see Supplementary Fig. 3), corresponding to two asymmetric CO stretching vibrations can be observed. The general structure was confirmed with single X-ray crystal analysis. Minor scrambling is observed with Cl^−^ bond to Ru, i.e. replacement of Br^−^. In a similar fashion the counterion distributed 1:1 (Br:Cl), giving an overall molecular sum formula of C_21_H_25_Br_0.63_Cl_1.29_N_5_O_2_Ru. The major halide bond to the metal centre was chloride, as confirmed with ESI-ms. The analysis gave a mass of 516*m*/*z* and a minor product with 562*m*/*z* (see Supplementary Fig. 4). The analogous complex consisting solely of Cl counterions was synthesised as a control experiment to rule out the influence of multiple halides. The exchange of counterions in **L-1** to Cl employing an ion exchange resin yielded the ligand **L-1(Cl)** (Supplementary Fig. 5), though at significantly reduced yields compared to **1** (see “Methods” section for more details). The formation of the carbene complex following the same procedure employed for the synthesis of **1**, yields **1-Cl** at significantly lower yield. The complex was characterised by ESI-ms, where **1-Cl** displayed a sole peak at 516*m*/*z* (see Supplementary Fig. 6), whereas identical NMR shifts were observed compared to **1**.

The single crystal analysis of **1** (Supplementary Fig. 7) indicated an average molecular mass of 578.11 g/mol, distributing to complexes with the sum formulas of C_21_H_25_Cl_2_N_5_O_2_Ru and C_21_H_25_BrClN_5_O_2_Ru. This in bromide to chloride ratio of 30:70. Indeed, the observed mass peaks for the M-Cl^+^ to M-Br^+^ of ca. 73:27 indicate that the ratio determined by single crystal analysis is valid. Further evidence for this ratio was found by ICP-OES analysis. Here a Ru content of 19.2 mg L^−1^ was found in a 0.199 mM solution (for 100% Cl expected Ru content is 20.8 mg L^−1^ and for 100% Br expected Ru content is 18.2 mg L^−1^), which indicate a molar ratio between Br^−^:Cl^−^ of 70:30. In this way a molecular mass of 578.11 g/mol was used to determine the amount of catalyst in solution. Furthermore, neither the addition of Cl^−^ or Br^−^ showed a difference in catalytic activity or stability in comparison to the non-modified system, indicating that the halide plays an insignificant role onto the catalytic activity.

### Catalytic efficiency evaluation

In an initial screening of catalytic activity, it was observed that **1** (0.28 mM) achieves a concentration of 0.5 M solution of FA at 80 °C in DMSO:water (5 v/v% water) in the presence of 1-butyl-2,3-dimethylimidazolium acetate (BMMI.OAc) under 60 bar pressure (H_2_:CO_2_ = 1:1). Furthermore, in the absence of catalyst we do not observe any conversion under elsewise identical reaction conditions (6 mL DMSO:H_2_O, 3.3 mmol BMMI.OAc, with *P*_H2_ = *P*_CO2_ = 30 bar at 80 °C). According to the thermodynamic parameters previously calculated for this system^16^, the concentration of FA observed is close to the thermodynamic equilibrium at ~120 °C (see Supplementary Fig. 8). Above 80 °C the decomposition of DMSO was observed. Fortunately, when BMMI.OAc is employed as an additive the reaction can be performed in a broad range of solvents^16^. In dioxane:H_2_O the catalytic system achieves similar concentration of FA at 80 and 100 °C as in DMSO at 80 °C (0.5 M at 100 °C in dioxane:H_2_O and 0.5 M in DMSO:H_2_O). Note also that the temperature range employed did not lead to a decomposition of the IL, as evidenced by NMR spectroscopy provided in Supplementary Fig. 9.

In order to investigate the thermal stability of the catalyst we investigated the reaction mixture towards the formation of nanoparticles during the reaction. It is important to note that no evidence of nanoparticle formation was observed under the reaction conditions assayed, even when a relatively high concentration of catalyst (0.28 mM) was employed. Even at high temperatures and pressures (120 °C, 60 bar (*P*_H2_ = *P*_CO2_)), TEM and DLS analysis showed no evidence of nanoparticles (see ESI for more details). Furthermore, the catalyst was not active in a reaction typically catalysed by Ru nanoparticles^27–31^, such as the hydrogenation of benzene at 120 °C under 50 bar H_2_ in the presence of BMMI.OAc even when high catalyst loadings are used, see SI for more details.

In the next step the catalyst activity and stability were explored. For this purpose, we lowered the concentration of catalyst (from 0.28 mM to 2.8 µM) and measured the TOF_ini_ after 4 h of reaction as an average value. We found that increasing the temperature led to an increase in the measurable TOF_ini_ after 4 h, at the expense of a lower achievable concentration of FA (and consequently TON) at temperatures above 120 °C (see Table 1 entries 5 and 6). This is due to the thermodynamic limitations of the system as depicted in Supplementary Fig. 8 in the Supporting information.

**Table 1:** Temperature-dependant results obtained with catalyst **1**.


Entry	Catalyst	*T* (°C)	[FA] (M)	TON^a^	TOF_ini_^b^ (h^−1^)
1	**1**	100	0.22 (0.01) ± 0.005	77,550 ± 1600	1080 ± 400
2	**1**	110	0.29 (0.02) ± 0.005	100,580 ± 1600	1750 ± 400
3^c^	**1**	120	0.49 (0.05) ± 0.005	162,900 ± 1600	4360 ± 400
4	**1**	130	0.40 (0.12) ± 0.005	141,000 ± 1600	10,620 ± 400
5	**1**	140	0.29 (0.19) ± 0.005	100,862 ± 1600	16,500 ± 400
6	**1-Cl**	120	0.49 (0.05) ± 0.005	163,000 ± 1600	4200 ± 400

*Reaction conditions***:** 6 mL dioxane;water (5 v/v% water), 3.3 mmol BMMI.OAc, 2.8 µM catalyst.

^a^Determined after 72 h.

^b^Determined after 4 h of reaction time.

^c^Data reproduced two times, error values within the error margin of the NMR determination, which were used as the standard deviation.

Interestingly, the nature of the halide in **1**, does not influence the catalytic activity observed. The compound **1-Cl** was tested under identical conditions than **1** as a control experiment, which showed the same catalytic activity in terms of TON and TOF (Table 1 entries 4 and 6). This suggests that the halide coordinated to the active Ru centre represents the leaving group and the catalytic activity is mainly influenced by the carbene pincer ligand employed.

**Fig. 3:**
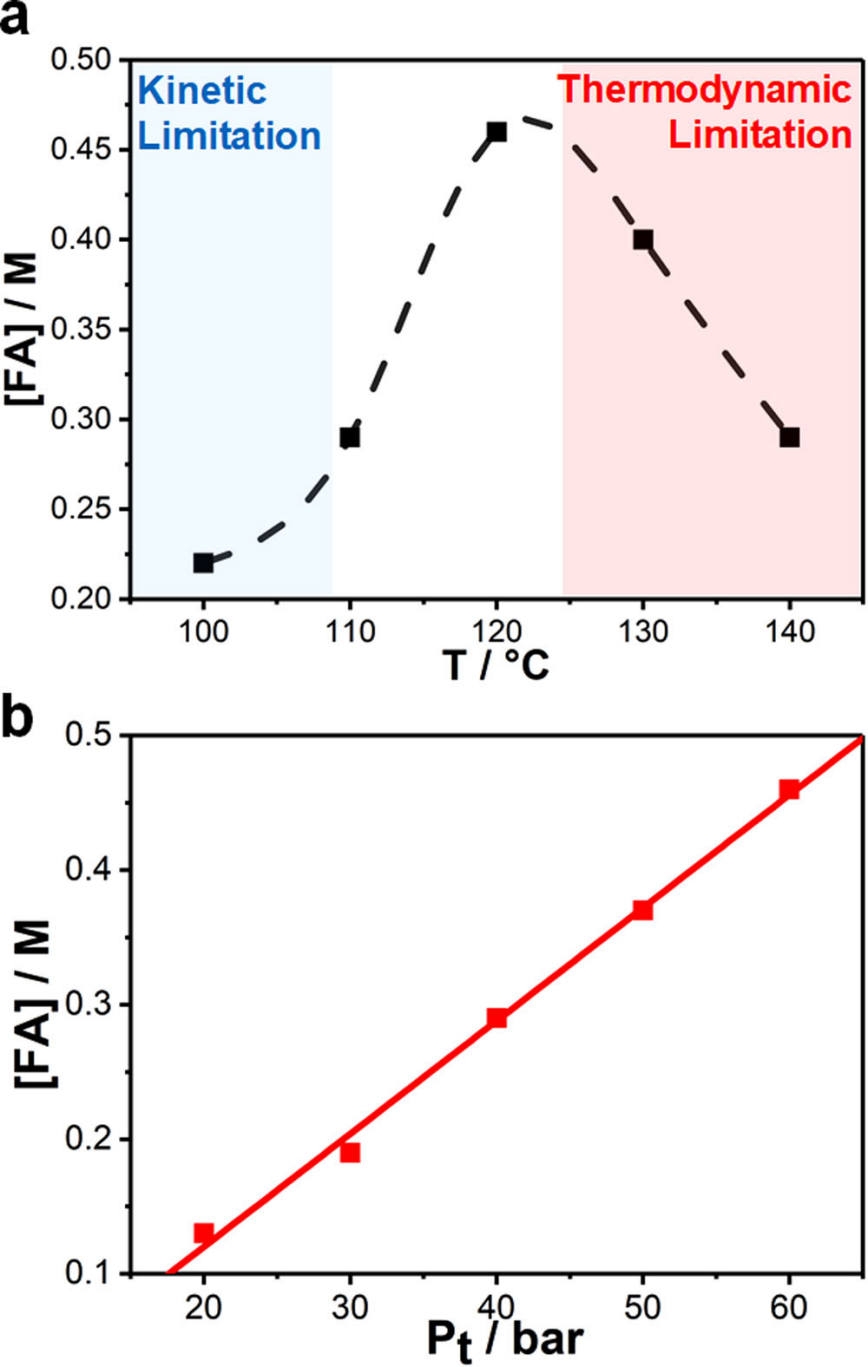
Effect of temperature and pressure on concentration of formic acid synthesised. **a** Optimal catalytic performance as a function of temperature, where kinetic and thermodynamic limitations are observed. **b** Concentration of FA observed as a function of the total gas pressure.

Plotting the concentration of FA generated after 72 h (Fig. 3a) allows for a better understanding of the efficiency of the catalytic system. Here a maximum concentration of FA can be observed at 120 °C. Below that temperature, the system is controlled by kinetic limitations of the catalyst, whilst at higher temperatures the reaction is limited by approaching the thermodynamic equilibrium inherent to the reaction under the studied conditions. Our previous report shows the thermodynamic equilibrium curve calculated employing Van’t Hoff plots for the CO_2_ hydrogenation to FA employing BMMI.OAc as a buffering system (see Supplementary Fig. 8)^16^.

In the next step the effect of pressure was investigated. Here a linear correlation in between the achieved concentration of FA and pressure can be observed (Fig. 3b). This is in consistence with the Henry’s law, which predicts a linear relationship between the amount of dissolved gas and the pressure.

**Fig. 4:**
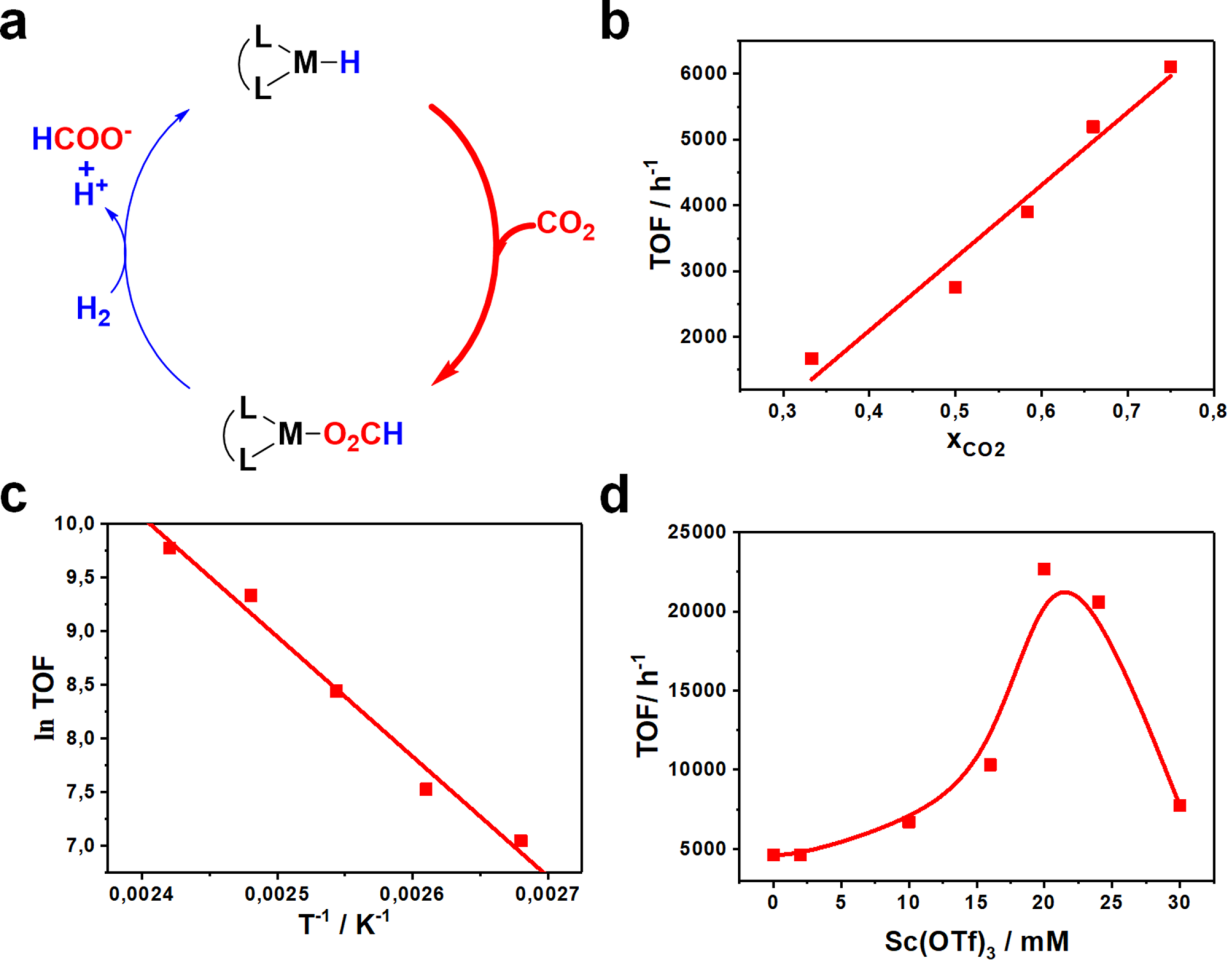
Analysis of rate determining step and conditions on catalytic activity observed. **a** Simplified reaction mechanism with the rate-determining step (CO_2_ insertion) highlighted. **b** Kinetic dependence of transformation on the partial pressure of CO_2_. **c** Arrhenius plot for the IL-buffered FA synthesis. **d** Effect of Sc(OTf)_3_ on observed catalyst activity.

Mechanistically, the hydrogenation of CO_2_ to FA can be divided into two main steps. In the first step, a hydride is transferred onto CO_2_. In the second step, H_2_ is activated releasing a proton and formate, with a simultaneous regeneration of the active hydride (Fig. 4a). In order to determine the rate-determining step, various gas compositions were tested (maintaining constant the total pressure). The observed reaction rate decreased significantly at low partial pressure of CO_2_ (Fig. 4b). Indeed, at 20 bar CO_2_ and 40 bar H_2_ a TOF_ini_ of 2700 h^−1^ was obtained. On the contrary an increased partial pressure of CO_2_ led to increased rates, i.e. at 45 bar CO_2_ and 15 bar H_2_ a TOF_ini_ of 9700 h^−1^ is determined, (cf. 4360 h^−1^ at *P*_CO2_ = *P*_H2_ = 30 bar). These experiments clearly show a strong dependence on CO_2_, thus indicating the CO_2_ insertion to be the rate-determining step.

An activation energy of 93 ± 7 kJ mol^−1^ was determined from the Arrhenius plot (Fig. 4c). The activation energy can either be decreased by increasing the electron donation onto the metal, leading to a more nucleophilic hydride, thus decreasing the hydricity (${{\Delta }}G_{{\mathrm{{H}}}^ - }$)^32^. Based on our recent study^21^, a low hydricity will ultimately lead to a more nucleophilic/basic hydride, rendering the catalyst prone protonation and hence deactivation.

It has been demonstrated that charged intermediates and transition state formed during the CO_2_ insertion can be stabilised with Lewis acids (see Supplementary Fig. 10 for a suggested mechanism)^33–36^. Therefore Sc(OTf)_3_ was tested due to its high solubility in organic solvents, tolerance towards water and due to its relatively weak acidic nature^37^. It was found that Sc(OTf)_3_ significantly increased the reaction rate. However, smaller amounts have only a minor effect on the rate (Fig. 4d). At an optimal concentration of 20 mM for Sc(OTf)_3_ was found a TOF_ini_ of 21,200 h^−1^, which corresponds to 4.9 times the activity without using the lewis acid. Above this concentration a drop in rate was observed, presumably due to catalyst poisoning as already observed in previous studies^38^. However, in the presence of Sc(OTf)_3_ the concentration of FA generated decreased slightly to 0.32 M, yielding a TON of 114,400 (Table 2, entry 2). Increasing the hydrogen to carbon dioxide ratio did not show any pronounced effects on activity nor stability (Table 2, entry 3). Under these conditions, the amount of catalyst was reduced to 1.7 nmol, which led to an unprecedented TON of 833,800, while the TOF_ini_ was maintained at 20,600 h^−1^ (Table 3, entry 4). In this case, there is no conversion without BMMI.OAc or with Sc(OTf)_3_ only as catalyst.

**Table 2:** Optimised conditions for the hydrogenation of CO_2_ to FA.


Entry	1 (µM)	*P*_H2_:*P*_CO2_ (bar:bar)	[Sc(OTf)_3_] (mM)	[FA] (M)	TON	TOF_ini_ (h^−1^)
1	2.83	30:30	0	0.46 ± 0.005	162,900 ± 1600	4360 ± 400
2	2.83	30:30	20	0.32 ± 0.005	114,400 ± 1600	21,200 ± 400
3	2.83	45:15	20	0.36 ± 0.005	126,000 ± 1600	22,000 ± 400
4^a^	0.28	45:15	20	0.26 ± 0.005	833,800 ± 1600	20,600 ± 400

*Reaction conditions*: 2.83 µM catalyst, *T* = 120 °C in 6 mL 1,4-dioxane:H_2_O (5 v/v% H_2_O) in the presence of 3.3 mmol BMMI.OAc; TON and [FA] determined after 72 h using BMMI.OAc as an internal standard, TOF_ini_ determined after 4 h.

^a^0.28 µM, TON and [FA] determined after 5 days using BMMI.OAc as an internal standard, TOF_ini_ determined after 18 h.

It is worth noting the remarkable activity and stability of our catalytic system, which shows ca. 50 times higher value in TON and 23 times higher value in TOF_ini_ in the hydrogenation of CO_2_ to FA under buffering conditions than the best reported to date^9–11,14,16^. Furthermore, the TON and TOF_ini_ towards FA are comparable to values obtained under basic conditions^39–41^. Interestingly, the CNC–Ru pincer reported here outperforms its previously reported analogue with a six-membered bite angle ring, even though the previous complex was reported to operate under basic conditions^42^.

To conclude, we have presented a catalytic system capable of highly efficiently transform CO_2_ into FA under buffering conditions. The combination of basic ionic liquids, highly robust catalysts based on Ru pincer N-heterocyclic carbenes and Lewis acid catalysts showed high efficiency at elevated temperatures, balancing kinetic and thermodynamic performance. The catalytic activity observed is the highest observed to date for base-free systems and comparable to literature reports employing basic conditions that lead to the formation of thermodynamically stable formate salts. This work will open new avenues in the hydrogenation of CO_2_ to chemicals and for the storage of hydrogen in liquid energy vectors.

## Methods

### General information

The ionic liquids 1,2-dimethyl-3-butylimidazolium acetate (BMMI.OAc) were prepared from literature methods^43^. [Ru(Cl)_2_(CO)_2_]_*n*_ complex was synthesised according to a reported method^44^. 1,2-dimethylimidazole was purchased from IOLITEC while *n*-chlorobutane, dimethylsulfoxide, Sc(OTf)_3_ and deuterated dimethylsulfoxide were obtained from Sigma Aldrich. Dioxane and ethylene glycol was provided by VWR. RuCl_3_·*x*H_2_O was provided by Precious Metals Online. All the ILs were placed under vacuum at 50 °C for 2–3 h prior to use. NMR measurements were performed on a 400 MHz Bruker or 500 MHz Bruker with *d*1 = 50 s and ESI-MS analysis was performed on a Bruker microTOFII.

### Synthesis of 2,6-bis(1-butylimidazolium)pyridine dibromide

2,6-bis(1-butylimidazolium)pyridine dibromide was prepared according to a previously reported method^45^. 3.36 g (27 mmol, 2 eq) 1-butylimidazole and 3.2 g (13.5 mmol, 1 eq) 2,6-dibromopyridine are stirred at 150 °C for 72 h under argon. After cooling the reaction mixture to room temperature, the reaction mixture was dissolved in CHCl_3_ and precipitated with Et_2_O. The desired product is isolated as a white solid after repetitive precipitation from MeOH with Et_2_O. Yield: 4.1 g (8.37 mmol, 62%). ^1^H-NMR and ^13^C spectra match previously reported results^43^.

### Synthesis of 2,6-bis(1-butylimidazolium)pyridine dichloride

2,6-bis(1-butylimidazolium)pyridine dichloride was prepared by using amberlite 402 (Cl form) ion exchange resin. 2 g (4.1 mmol) 2,6-bis(1-butylimidazolium)pyridine dibromide was dissolved in a small amount of water and passed through an ion exchange column containing 50 g amberlite 402 (Cl form) ion exchange resin using water as the eluent. The solvent was removed under reduced pressure and the product is obtained upon dissolving the crude product in 5 mL MeOH and precipitation with Et_2_O. The product is obtained as a white powder.

Yield: 1 g (2.5 mmol, 61%)

### Synthesis of Ru–CNC pincer

In the dark under argon: 700 mg (1.55 mmol, 1 eq) 2,6-bis(1-butylimidazolium)pyridine dibromide and 400 mg (1.73 mmol, 1.12 eq) Ag_2_O are dissolved in 10 mL dry and degassed DCM. The reaction mixture is stirred in the dark under an argon atmosphere for 24 h. Afterwards the solution is filtered through a celite pad, the solvent is removed in vacuum. The resulting white solid is washed with 3 × 15 mL Et_2_O. The resulting white solid is directly used for the next step.

908 mg of the white precipitate obtained in the previous step and 300 mg (1.30 mmol, 1 eq) [Ru(CO)_2_(Cl)_2_] are mixed in 20 mL THF and the suspension is stirred under argon in the dark for 48 h at 55 °C. The resulting reaction mixture is cooled to room temperature and filtered through a short celite pad and reduced under vacuum. The resulting yellow solid is re-dissolved in 5 mL MeOH and filtered. The product is obtained upon precipitation with Et_2_O and finally washed with MeCN. Crystals are obtained by slow diffusion of Et_2_O into MeOH (see supplementary information for more details on the crystal structure). Yield: 350 mg (0.61 mmol, 47%). CHN: found: 11.3% N, 42.0% C, 4.7% H; expected: 12.1% N, 43.6% C, 4.4% H. Sum formula C_21_H_25_Br_0.6_Cl_1.4_N_5_O_2_Ru. ^1^H-NMR (DMSO-d6, 400 MHz): *δ* 8.67 ppm (d, ^3^*J* = 2.24 Hz, 2H), 8.62 ppm (t, ^3^*J* = 8.2 Hz, 1H), 8.18 ppm (d, ^3^*J* = 8.24 Hz, 2H), 7.91 ppm (2 H, ^3^*J* = 2.20 Hz, 2H), 4.52–5.13 ppm (m, 4H), 1.89 ppm (p, ^3^*J* = 7.4 Hz, 4H), 1.58–1.22 ppm (m, 4H), 0.95 ppm (t, ^3^*J* = 7.4 Hz, 6H). ^13^C-NMR (DMSO-d6, 126 MHz): *δ* 181, 151, 125, 120, 119, 109, 52, 33, 20, 14 ppm. ESI-ms: 592*m*/*z*. ATR-IR: *υ* [cm^−1^]: 3200 (C–H_arom_), 3000 (C–H_Im_), 2900 (C–H_al_), 2051 (C=O), 1993 (C=O).

### Synthesis of Ru–CNC pincer Cl form

In the dark under argon: 614 mg (1.55 mmol, 1 eq) 2,6-bis(1-butylimidazolium)pyridine dichloride and 400 mg (1.73 mmol, 1.12 eq) Ag_2_O are dissolved in 10 mL dry and degassed DCM. The reaction mixture is stirred in the dark under an argon atmosphere for 24 h. Afterwards the solution is filtered through a celite pad, the solvent is removed in vacuum. 300 mg (1.30 mmol, 0.8 eq) [Ru(CO)_2_(Cl)_2_] are mixed in 20 mL THF and the suspension is stirred under argon in the dark for 48 h at 55 °C. The resulting reaction mixture is cooled to room temperature and filtered through a short celite pad and reduced under vacuum. The resulting yellow solid is re-dissolved in 5 mL MeOH and filtered. The product is obtained upon precipitation with Et_2_O and finally washed with MeCN. Yield: 52 mg (0.09 mmol, 6%). Sum formula C_21_H_25_Cl_2_N_5_O_2_Ru. ^1^H-NMR (DMSO-d6, 400 MHz): *δ* 8.67 ppm (d, ^3^*J* = 2.24 Hz, 2H), 8.62 ppm (t, ^3^*J* = 8.2 Hz, 1H), 8.18 ppm (d, ^3^*J* = 8.24 Hz, 2H), 7.91 ppm (2 H, ^3^*J* = 2.20 Hz, 2H), 4.52–5.13 ppm (m, 4H), 1.89 ppm (p, ^3^*J* = 7.4 Hz, 4H), 1.58–1.22 ppm (m, 4H), 0.95 ppm (t, ^3^*J* = 7.4 Hz, 6H). ^13^C-NMR (DMSO-d6, 126 MHz): *δ* 181, 151, 125, 120, 119, 109, 52, 33, 20, 14 ppm. ESI-ms: 516*m*/*z*.

## Supplementary information

Supplementary Information

## Data Availability

The datasets generated during and/or analysed during the current study are available from the corresponding author on reasonable request. Source data are provided with this paper.

## Source data

Source Data
