# Supplementary material for: Efficient carbon dioxide hydrogenation to formic acid with buffering ionic liquids
Source: Nat Commun. 2021 Jan 11;12:231. doi: 10.1038/s41467-020-20291-0 (PMC7801478; doi:10.1038/s41467-020-20291-0)
Supplement: Supplementary file 1 — Supplementary Information [file 41467_2020_20291_MOESM1_ESM.pdf]

Efficient carbon dioxide hydrogenation to formic acid with buffering ionic liquids

Andreas Weilhard,[a] Stephen P. Argent,[b] Victor Sans* [a,c]

[a] Faculty of Engineering, University of Nottingham, Nottingham, NG7 2RD, United Kingdom

[b] School of Chemistry, University of Nottingham, Nottingham, NG7 2RD, United Kingdom

[c] Institute of Advanced Materials (INAM), Universitat Jaume I, Castellon, 12071, Spain

Supplementary tables

Supplementary Table 1: Summary of relevant homogeneous CO_2-hydrogenation systems to prepare free formic acid (HCO_2H) by different catalysts from literature. Adapted from Ref. 7.

Entry	Catalyst	[FA] / M	TON	TOF / h^{-1}	Ref.
1	$[Ru_2Cl_2(PTA)_4]$	1.9	749	7.34	1
2	$[Ru(Acriphos)(PPh_3)(Cl)(PhCO_2)]$	1.27	16310	1019	2
3	$[(\eta^6C_6Me_6)Ru^{II}(L)(OH_2)SO_4]$	-	55	-	3
4	$[(CpIr^{III}(L)(OH_2)]$	-	18	-	4
5	$[RhCl(mtppms)_3]$	0.13	-	-	5
6	$[Ru(cod)(methally)_2]$	0.54	599	314	6
7	$[Ru_3(CO)_{12}]$	1.2	17000	102	7
**8**	**[1]**	**0.49**	**162900**	**4360**	**This work**
**9**	**[1], $Sc(OTf)_3$**	**0.26**	**833800**	**20600**	**This work**

Supplementary Table 2 catalyst comparison and poisoning experiments

Entry	Catalyst	Additive (mmol)	[FA]$^{a)}$ / M	TON$^{a)}$	TOF$^{b)}$ / h^{-1}
1	1	-	0.50	1763	58
2	1	NaCl (0.12 mmol)	0.50	1763	63
3	1	NaBr (0.12 mmol)	0.50	1763	56

Reaction conditions 1.6 µmol catalyst, 3.3 mmol BMMI.OAc, $P(H_2) = P(CO_2)$ = 30 bar, 6 ml DMSO:H_2O (5 v/v% H_2O), T = 80°C, a) determined after 72h, b) determined after 4h.

Supplementary Table 3 Calculation of catalyst amounts and concentrations in the consecutive catalytic reaction.

Entry	Stock cat. Solution / mM	Aliquot for reaction / mL	Cat. in reaction mixture / µmol	Conc. cat in reaction mixture/ µM
1	0.17	1 ± 0.01	0.17	28.3
2	0.017	1 ± 0.01	0.017	2.83
3	0.0017	1 ± 0.01	0.0017	0.28

Supplementary Table 4 Effect of water content onto the hydrogenation of CO_2 to FA.

Entry	Water content / %	t / h	Conc. FA[a] / M	TON[a]	TOF[b] / h^{-1}
1	5	4	0.05	17500	4360
2	20	4	0.05	16600	4150
3	50	4	0.02	5800	1460
4	80	4	0.01	2000	500
5	30	4	0.03	11600	2900
6	5	72	0.46	162900	2260
7	20	72	0.36	127900	1780

Reaction conditions catalyst 0.017 µmol, T = 120 °C, 3.3 mmol BMMI.OAc, 6 mL solvent; a) determined after 72h, b) determined after 4h.

Supplementary Table 5 Crystal data and structure refinement for **1.**

Identification code	**RUASWB**
Empirical formula	$C_{24.5}H_{29}Br_{0.63}Cl_{1.29}N_5O_2Ru$
Formula weight	622.38
Temperature/K	120(2)
Crystal system	monoclinic
Space group	C2/m
a/Å	8.9075(3)
b/Å	13.6660(4)
c/Å	22.4967(7)
α/°	90
β/°	97.948(3)
γ/°	90
Volume/Å^3	2712.21(15)
Z	4
ρ_{calc}g/cm^3	1.524
μ/mm^{-1}	7.172
F(000)	1259.0

Crystal size/mm^3	0.397 × 0.194 × 0.071
Radiation	CuKα (λ = 1.54184)
2Θ range for data collection/°	7.936 to 149.108
Index ranges	-10 ≤ h ≤ 10, -16 ≤ k ≤ 16, -21 ≤ l ≤ 27
Reflections collected	10836
Independent reflections	2843 [R_{int} = 0.0387, R_{sigma} = 0.0241]
Data/restraints/parameters	2843/236/226
Goodness-of-fit on F^2	1.097
Final R indexes [I>=2σ (I)]	R_1 = 0.0338, wR_2 = 0.0840
Final R indexes [all data]	R_1 = 0.0340, wR_2 = 0.0841
Largest diff. peak/hole / e Å^{-3}	1.64/-0.95

Supplementary Table 6 Fractional Atomic Coordinates (×10^4) and Equivalent Isotropic Displacement Parameters (Å^2×10^3) for RUASWB. U_{eq} is defined as 1/3 of of the trace of the orthogonalised U_{IJ} tensor

Atom	*x*	*y*	*z*	U(eq)
Ru1	3063.9(3)	5000	2037.6(2)	19.13(11)
Cl1	5400(6)	5000	2721(4)	23.4(7)
O2A	1219(5)	5000	3059.7(17)	48.4(9)
O2B	124(4)	5000	1188.1(16)	37.3(7)
N22	4304(2)	3350.7(16)	1457.8(10)	19.5(4)
N25	3082(2)	2635.2(17)	2088.9(10)	20.1(5)
N11	4405(3)	5000	1364.2(14)	19.8(6)
C12	4882(3)	4151.1(19)	1165.4(11)	19.7(5)
C1B	1217(4)	5000	1471(2)	23.4(8)
C13	5881(3)	4112(2)	748.5(12)	23.4(5)
C14	6375(5)	5000	544.9(17)	26.1(8)
C21	3419(3)	3521.4(19)	1899.5(11)	19.2(5)
C23	4489(3)	2353(2)	1369.7(12)	22.1(5)
C24	3729(3)	1911(2)	1769.1(12)	23.4(5)
C31	2293(3)	2450(2)	2608.2(12)	22.6(5)
C1A	1912(5)	5000	2686(2)	32.0(9)
C32	3405(3)	2364(2)	3182.5(12)	27.1(6)
C33	2605(3)	2226(3)	3730.7(13)	32.9(7)
C34	3720(4)	2109(3)	4303.1(14)	45.5(9)
Br2	1686.0(13)	3328.7(10)	273.8(6)	50.3(5)
Cl2	1686.0(13)	3328.7(10)	273.8(6)	50.3(5)
C7S	2260(30)	4940(60)	4582(8)	89(9)
C1S	3910(20)	5010(40)	4860(5)	61(4)
C2S	5030(20)	5010(60)	4508(10)	74(6)
C3S	6510(30)	5090(60)	4757(10)	77(9)
C4S	6870(30)	5120(30)	5369(9)	63(8)
C5S	5750(20)	5130(30)	5723(7)	51(8)
C6S	4262(18)	5080(60)	5469(7)	45(7)
Br1	5648(16)	5000	2768(9)	23.4(7)

Supplementary Table 7 Anisotropic Displacement Parameters (Å^2×10^3) for 1. The Anisotropic displacement factor exponent takes the form: $-2\pi^2[h^2a^{*2}U_{11}+2hka^*b^*U_{12}+\ldots]$.

Atom	U_{11}	U_{22}	U_{33}	U_{23}	U_{13}	U_{12}
Ru1	19.53(16)	13.58(16)	25.85(17)	0	8.71(11)	0
Cl1	22(2)	18.5(4)	28.0(14)	0	-3.3(13)	0
O2A	57(2)	43(2)	53(2)	0	32.9(18)	0
O2B	33.6(15)	24.2(16)	51.8(19)	0	-2.4(13)	0
N22	21.1(10)	15.2(10)	22.3(10)	-0.7(8)	2.7(8)	0.8(9)
N25	19.2(10)	15.0(11)	25.7(11)	1.3(8)	1.6(8)	-1.1(8)
N11	19.0(15)	19.2(15)	21.8(15)	0	4.7(12)	0
C12	19.0(12)	18.9(13)	20.3(12)	-0.8(10)	-0.1(9)	0.4(10)
C1B	19.3(15)	7.9(16)	45(2)	0	10.2(14)	0
C13	23.9(13)	23.8(14)	22.8(12)	-1.5(10)	4.5(10)	3.7(11)
C14	25.1(19)	33(2)	22.1(18)	0	9.0(15)	0
C21	17.0(11)	16.3(12)	24.1(12)	-0.5(10)	1.9(9)	0.5(10)
C23	24.8(12)	17.1(13)	23.5(12)	-3.3(10)	-0.4(10)	3.2(10)
C24	25.6(13)	14.3(12)	28.6(13)	-2.0(10)	-2.0(10)	1.3(10)
C31	19.0(11)	19.6(13)	29.5(13)	3.8(10)	4.5(10)	-2.3(10)
C1A	34(2)	21(2)	42(2)	0	9.5(18)	0
C32	19.6(12)	33.6(16)	28.6(13)	2.1(12)	4.5(10)	-1.3(12)
C33	24.0(13)	43.9(18)	31.7(15)	3.3(13)	7.1(11)	-0.7(13)
C34	38.0(17)	72(3)	26.9(15)	8.4(16)	5.7(13)	-0.3(18)
Br2	37.7(7)	59.1(9)	55.6(8)	20.1(6)	11.7(5)	-4.7(5)
Cl2	37.7(7)	59.1(9)	55.6(8)	20.1(6)	11.7(5)	-4.7(5)
C7S	123(13)	62(18)	74(11)	20(20)	-18(9)	-40(30)
C1S	111(11)	32(8)	39(6)	-8(17)	10(5)	-20(20)
C2S	140(14)	36(11)	52(8)	-10(20)	37(8)	-20(20)
C3S	128(13)	30(20)	87(10)	5(14)	54(9)	-13(19)
C4S	83(11)	20(20)	96(10)	-10(12)	32(8)	-13(13)
C5S	81(8)	20(20)	51(7)	-5(8)	13(5)	-1(9)
C6S	78(9)	27(19)	33(5)	-7(13)	18(5)	1(13)
Br1	22(2)	18.5(4)	28.0(14)	0	-3.3(13)	0

Supplementary Table 8 Bond Lengths for **1**.

Atom	Atom	Length/Å	Atom	Atom	Length/Å
Ru1	Cl1	2.410(5)	N11	C12[1]	1.334(3)
Ru1	N11	2.055(3)	N11	C12	1.334(3)
Ru1	C1B	1.937(4)	C12	C13	1.380(4)
Ru1	C21[1]	2.075(3)	C13	C14	1.390(3)
Ru1	C21	2.075(3)	C23	C24	1.342(4)
Ru1	C1A	1.895(5)	C31	C32	1.520(4)
Ru1	Br1	2.636(13)	C32	C33	1.519(4)
O2A	C1A	1.110(6)	C33	C34	1.522(4)
O2B	C1B	1.087(5)	C7S	C1S	1.52(2)
N22	C12	1.410(3)	C1S	C2S	1.358(17)
N22	C21	1.371(3)	C1S	C6S	1.366(17)
N22	C23	1.390(3)	C2S	C3S	1.36(3)
N25	C21	1.332(3)	C3S	C4S	1.37(2)
N25	C24	1.394(3)	C4S	C5S	1.357(18)
N25	C31	1.467(3)	C5S	C6S	1.37(2)

Supplementary Table 9 Bond Angles for **1**.

Atom	Atom	Atom	Angle/°	Atom	Atom	Atom	Angle/°
N11	Ru1	Cl1	86.1(2)	C12	N11	Ru1	119.45(16)
N11	Ru1	C21[1]	76.91(7)	C12[1]	N11	Ru1	119.45(16)
N11	Ru1	C21	76.91(7)	C12	N11	C12[1]	120.9(3)
N11	Ru1	Br1	85.0(5)	N11	C12	N22	111.4(2)
C1B	Ru1	Cl1	178.5(2)	N11	C12	C13	121.8(3)
C1B	Ru1	N11	92.43(15)	C13	C12	N22	126.8(2)
C1B	Ru1	C21[1]	91.98(7)	O2B	C1B	Ru1	174.8(4)
C1B	Ru1	C21	91.98(7)	C12	C13	C14	117.0(3)
C1B	Ru1	Br1	177.4(5)	C13[1]	C14	C13	121.6(4)
C21[1]	Ru1	Cl1	87.69(8)	N22	C21	Ru1	112.88(18)
C21	Ru1	Cl1	87.69(8)	N25	C21	Ru1	142.38(19)
C21[1]	Ru1	C21	153.66(14)	N25	C21	N22	104.7(2)
C21	Ru1	Br1	87.45(12)	C24	C23	N22	105.4(2)
C21[1]	Ru1	Br1	87.45(12)	C23	C24	N25	107.9(2)
C1A	Ru1	Cl1	91.2(3)	N25	C31	C32	111.3(2)
C1A	Ru1	N11	177.26(16)	O2A	C1A	Ru1	179.0(4)
C1A	Ru1	C1B	90.31(19)	C33	C32	C31	112.1(2)
C1A	Ru1	C21[1]	103.00(7)	C32	C33	C34	112.1(2)
C1A	Ru1	C21	103.00(7)	C2S	C1S	C7S	120.5(14)
C1A	Ru1	Br1	92.3(5)	C2S	C1S	C6S	119.9(19)
C21	N22	C12	119.3(2)	C6S	C1S	C7S	119.5(13)
C21	N22	C23	111.2(2)	C1S	C2S	C3S	120.5(19)
C23	N22	C12	129.5(2)	C2S	C3S	C4S	120(2)
C21	N25	C24	110.7(2)	C5S	C4S	C3S	120(2)
C21	N25	C31	124.5(2)	C4S	C5S	C6S	120.0(18)
C24	N25	C31	124.5(2)	C1S	C6S	C5S	119.8(15)

Supplementary Table 10 Torsion Angles for **1**.

A	B	C	D	Angle/°	A	B	C	D	Angle/°
Ru1	N11	C12	N22	2.0(3)	C23	N22	C12	C13	-5.5(4)
Ru1	N11	C12	C13	-174.8(2)	C23	N22	C21	Ru1	-178.73(17)
N22	C12	C13	C14	-176.1(3)	C23	N22	C21	N25	1.0(3)
N22	C23	C24	N25	0.6(3)	C24	N25	C21	Ru1	179.0(2)
N25	C31	C32	C33	177.0(3)	C24	N25	C21	N22	-0.6(3)
N11	C12	C13	C14	0.2(4)	C24	N25	C31	C32	83.4(3)
C12	N22	C21	Ru1	1.3(3)	C31	N25	C21	Ru1	-7.6(4)
C12	N22	C21	N25	-179.0(2)	C31	N25	C21	N22	172.8(2)
C12	N22	C23	C24	179.0(2)	C31	N25	C24	C23	-173.4(2)
C12[1]	N11	C12	N22	176.8(2)	C31	C32	C33	C34	178.3(3)
C12[1]	N11	C12	C13	0.0(5)	C7S	C1S	C2S	C3S	179(3)
C12	C13	C14	C13[1]	-0.4(6)	C7S	C1S	C6S	C5S	179(3)
C21	N22	C12	N11	-2.2(3)	C1S	C2S	C3S	C4S	3(5)
C21	N22	C12	C13	174.5(2)	C2S	C1S	C6S	C5S	-1(5)
C21	N22	C23	C24	-1.0(3)	C2S	C3S	C4S	C5S	-3(6)
C21	N25	C24	C23	0.0(3)	C3S	C4S	C5S	C6S	1(6)
C21	N25	C31	C32	-89.1(3)	C4S	C5S	C6S	C1S	1(6)
C23	N22	C12	N11	177.9(3)	C6S	C1S	C2S	C3S	-1(4)

Supplementary Table 11 Hydrogen Atom Coordinates (Å×10^4) and Isotropic Displacement Parameters (Å^2×10^3) for **1**.

Atom	*x*	*y*	*z*	U(eq)
H13	6215.49	3505.7	607.07	28
H14	7068.31	5000.01	259.51	31
H23	5037.55	2049.86	1086.61	27
H24	3645.84	1224.44	1824.26	28
H31A	1702.18	1836.29	2542.86	27
H31B	1575.41	2990.65	2649.05	27
H32A	4087.69	1801.66	3148.17	33
H32B	4034.15	2963.24	3234.43	33
H33A	1951.09	1638.11	3673.36	39
H33B	1946.48	2798.05	3772.31	39
H34A	4338.94	1522.9	4272.31	68
H34B	3161.09	2043.64	4646.72	68
H34C	4379.52	2685.39	4358.79	68
H7SA	2032.31	5449.43	4277.55	134
H7SB	1602.59	5022.92	4894.74	134
H7SC	2065.56	4293.85	4393.84	134
H2S	4780.54	4958	4084.52	88
H3S	7285.4	5120.49	4507.47	93
H4S	7903.09	5143	5545.4	76
H5S	6005.52	5165.82	6147.18	61
H6S	3479.38	5087.12	5716.35	54

Atom	*Occupancy*	Atom	*Occupancy*	Atom	*Occupancy*
Cl1	0.830(4)	Br2	0.188(4)	Cl2	0.312(4)
C7S	0.25	H7SA	0.25	H7SB	0.25
H7SC	0.25	C1S	0.25	C2S	0.25
H2S	0.25	C3S	0.25	H3S	0.25
C4S	0.25	H4S	0.25	C5S	0.25
H5S	0.25	C6S	0.25	H6S	0.25
Br1	0.170(4)				

Supplementary Figures

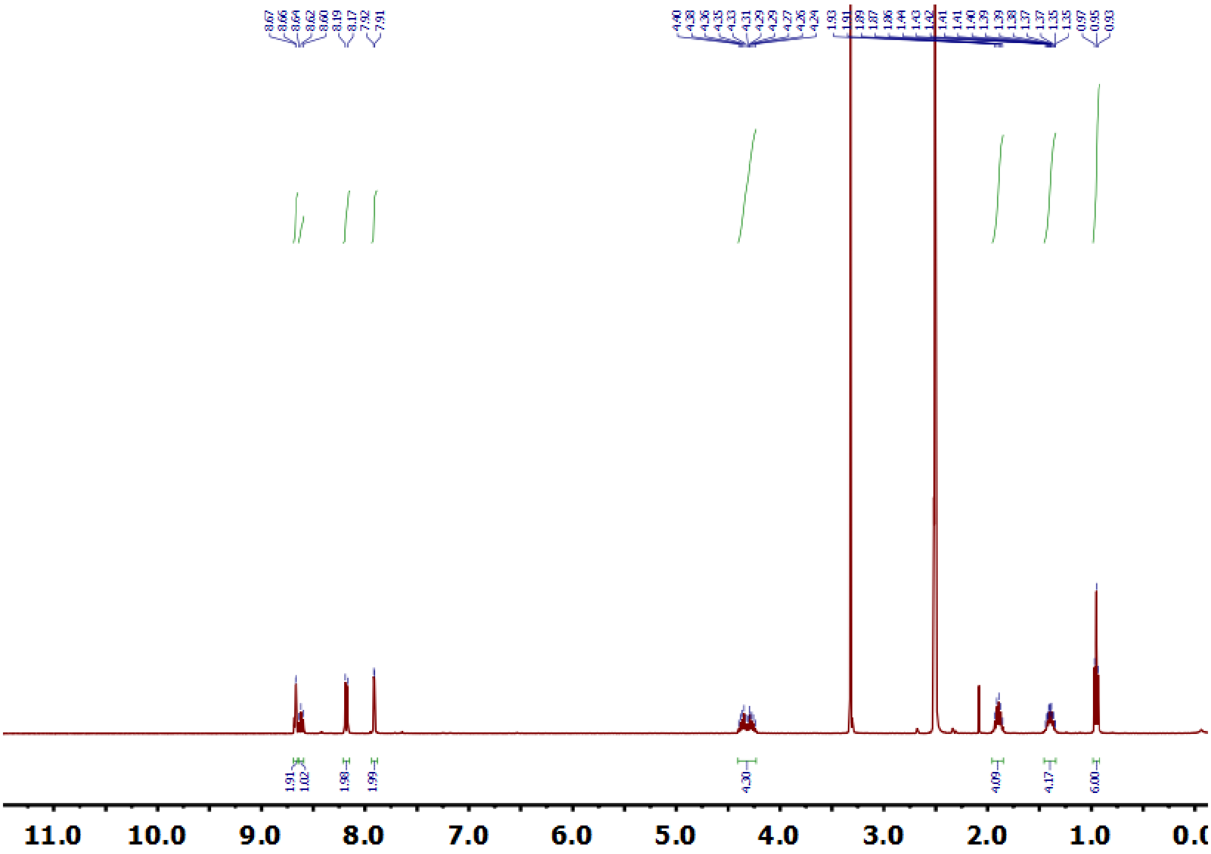

Supplementary Figure 1 ^{1}H NMR spectrum of complex **1** in DMSO-d6.

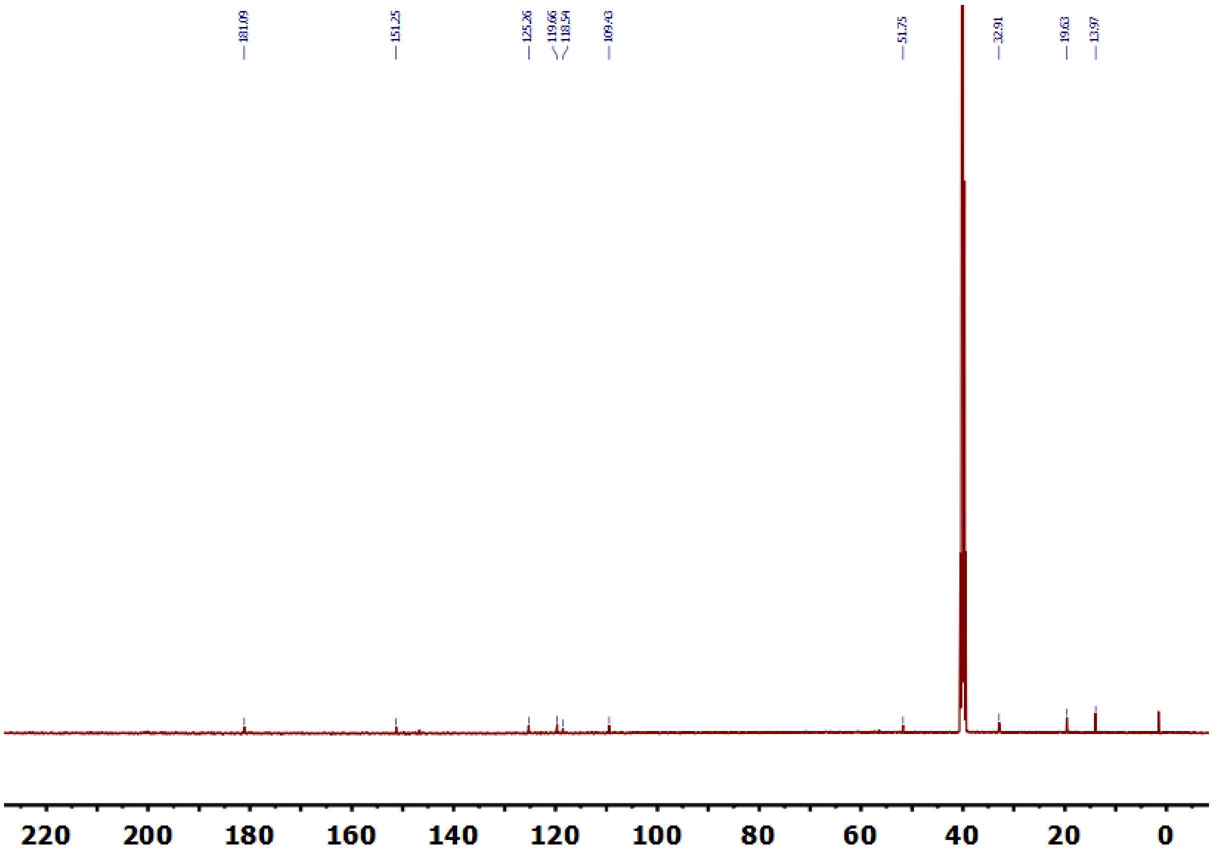

Supplementary Figure 2 13C NMR spectrum of complex **1** in DMSO-d6.

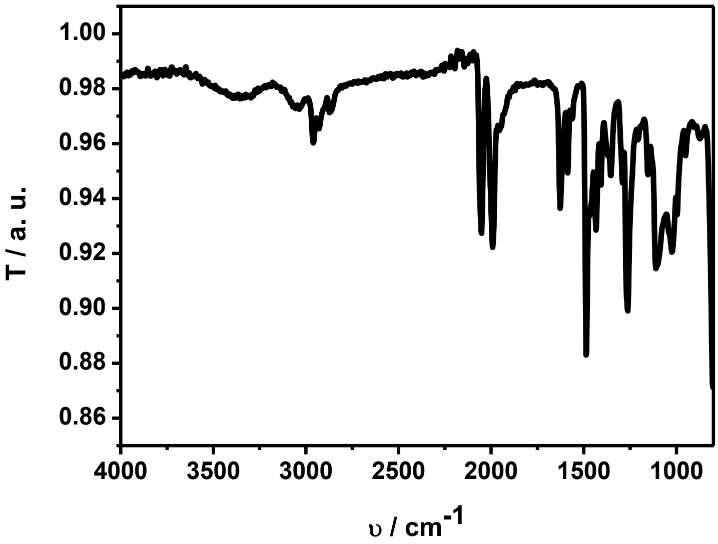

Supplementary Figure 3 ATR-Ir of complex **1**.

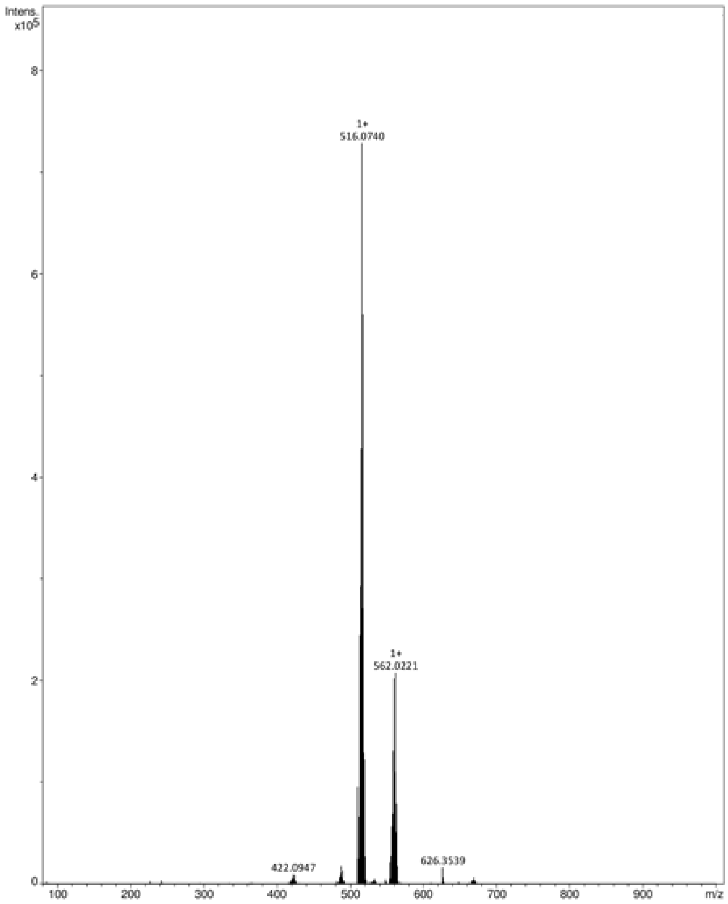

Supplementary Figure 4 ESI-ms spectrum of complex **1**.

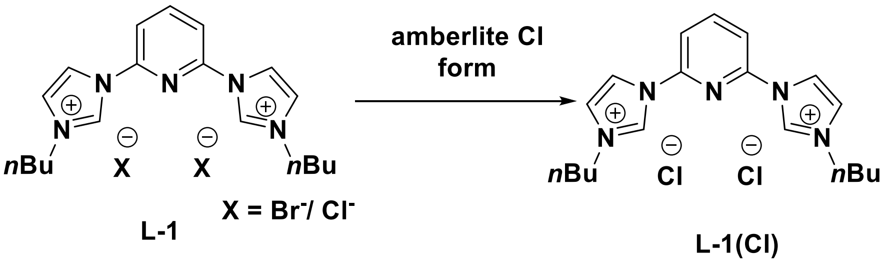

Supplementary Figure 5 Detail of synthetic strategy. Synthesis of ligand L-1(Cl) from L1 by anion exchange of Br^- by Cl^- employing an anion exchange resin.

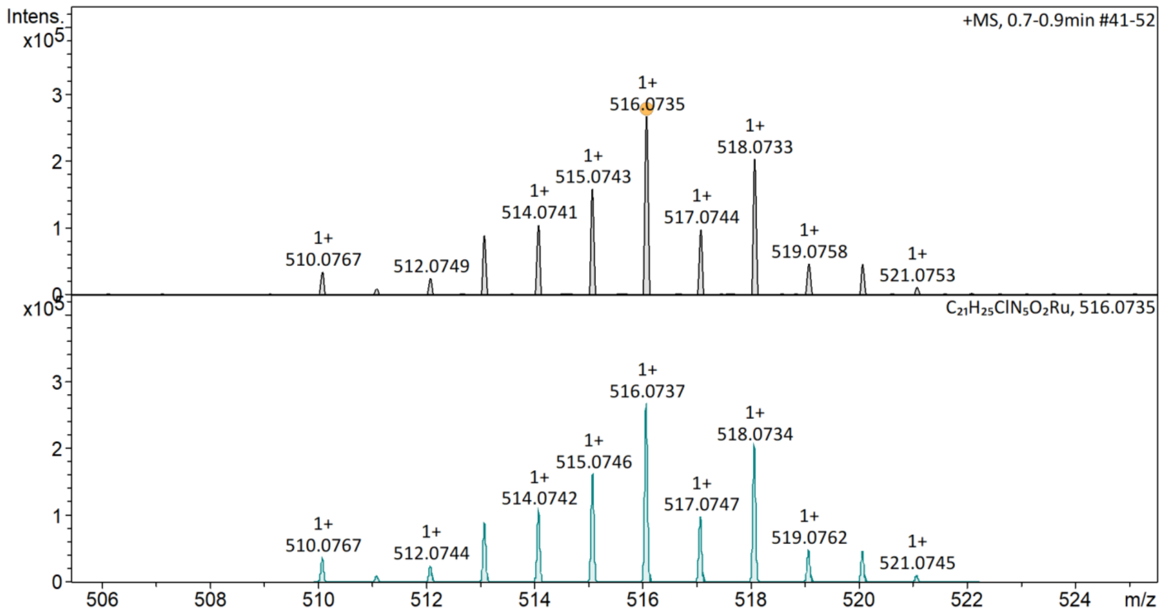

Supplementary Figure 6 ESI-ms spectrum of complex **1-Cl**.

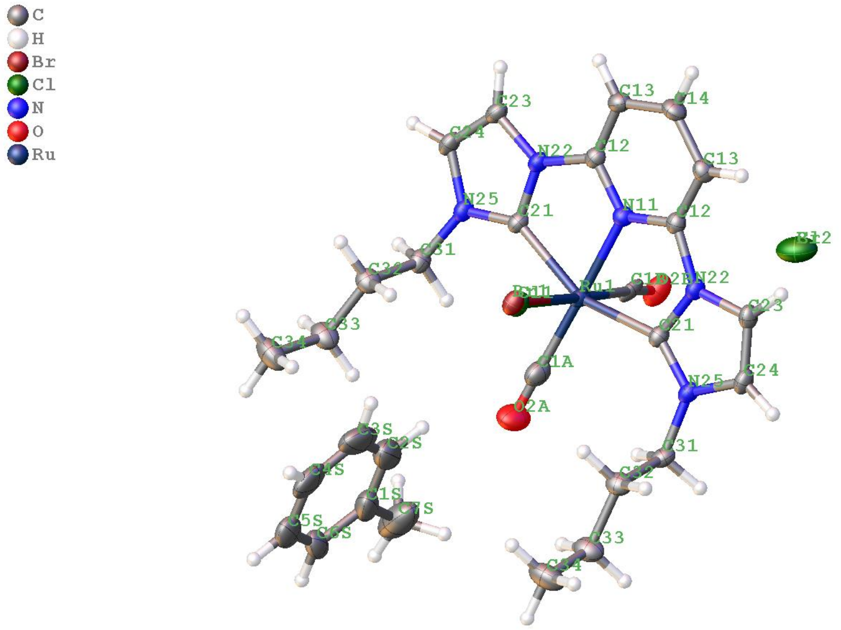

Supplementary Figure 7 Crystal structure of **1**.

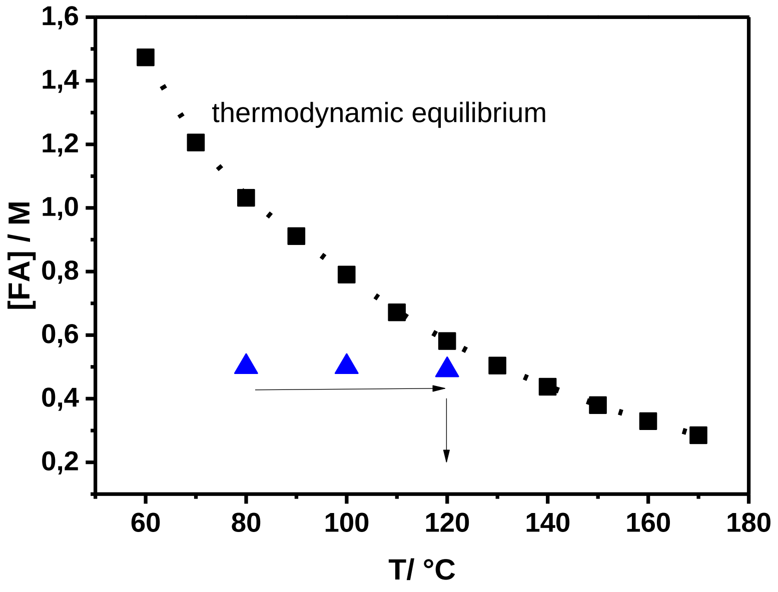

Supplementary Figure 8 Curve employed to predict optimal catalyst performance as a function of T, showing the thermodynamic equilibrium as a function of T under the experimental reaction conditions (black) and results from the initial screening (blue).

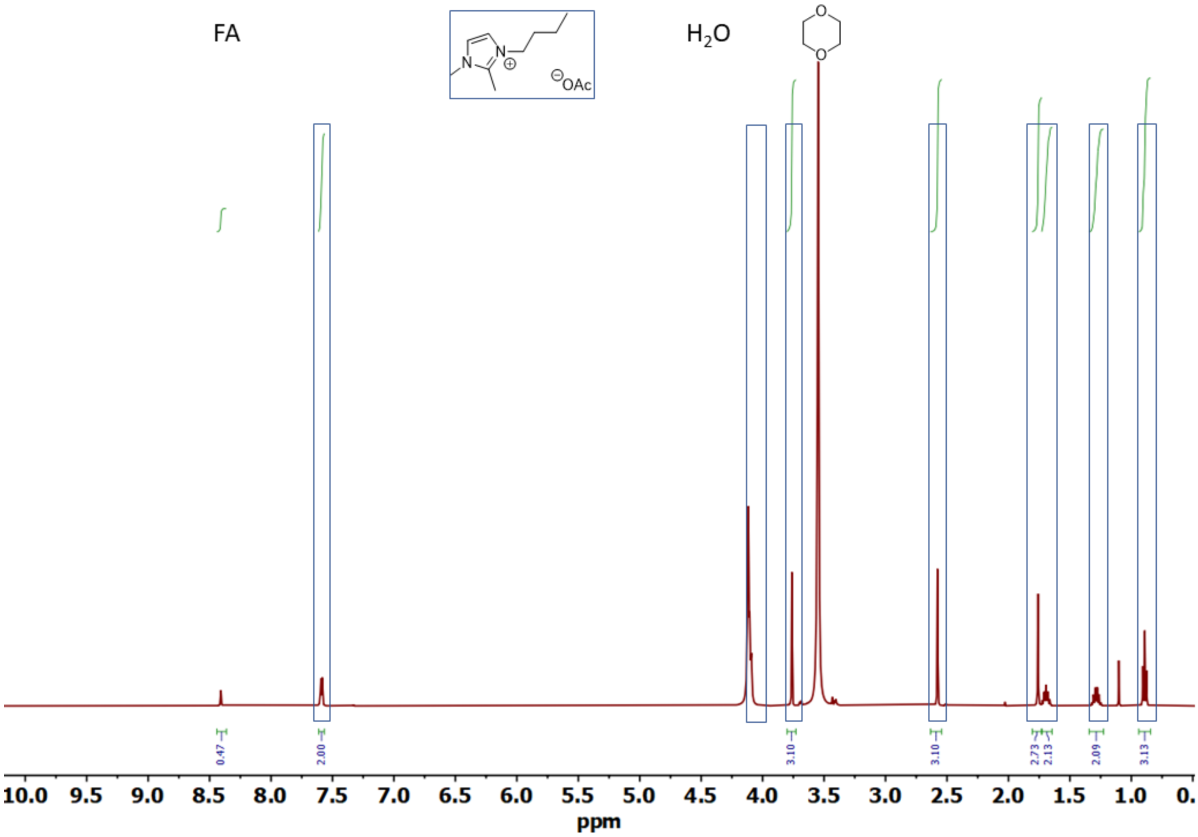

Supplementary Figure 9 *NMR spectrum obtained under 30 bar CO_2 and 30 bar H_2 with 1 after 18h at 140°C. Note that no decomposition of the IL is observed.*

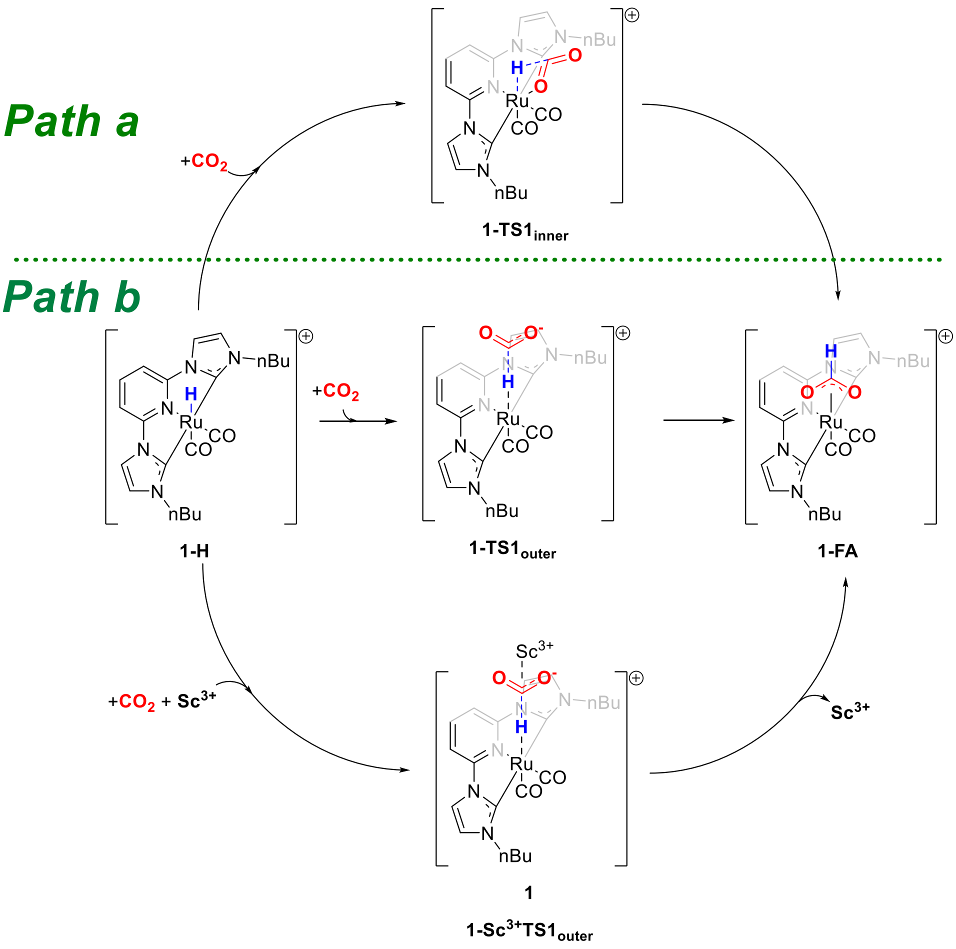

Supplementary Figure 10 Depiction of the insertion of CO_2 into a metal hydride bond formed by **1** (1-H). Path a depicts an inner sphere mechanism for the CO_2 insertion, path b) depicts the insertion of CO_2 via the outer sphere mechanism. The bottom pathway depicts a plausible stabilisation of a charged transition state.

Supplementary methods

ICP-OES measurement

1.15 mg (1.85 µmol) of **1** are dissolved in 10 mL of 2 v/v% HCl solution and the Ru content is measured with ICP-OES. Expected concentration of Ru was 18,6 mg L^{-1}. 19.2 mg L^{-1} was found confirming the expected formula obtained by other techniques.

Preparation of stock solutions

Solvent mixtures were prepared prior to utilization by mixing 95 mL ± 1 mL DMSO or 1,4-dioxane with 5 mL ± 0.2 mL deionized water (error values are according to the calibration uncertainty of the used measuring cylinder).

For the calculation of the amount of catalyst the average mass obtained by the crystal structure was used (622.23 g/mol). 1 mg (1.7 µmol) of **1** was dissolved in 10 mL ± 0.08 mL of the desired solvent system, giving a 0.17 mM catalyst stock solution. 1 mL ± 0.01 mL of this solution (0.17

mM) was diluted to 10 mL ± 0.08 mL in the same solvent system to give a 0.017 mM solution. 1 mL ± 0.01 mL of this solution (0.017 mM) was taken and diluted to 10 mL ± 0.08 mL in the same solvent system to give a 1.7 μM solution. (errors are given as the calibration uncertainties of the volumetric flask respectively micropipette).

$$dn = \sqrt{(dV \cdot C)^2 + \left(\frac{\sqrt{\left(\frac{\sqrt{\left(\frac{\sqrt{\left(\frac{dm}{MV_1}\right)^2 + \left(\frac{m \cdot dV_1}{M \cdot V_1^2}\right)^2}}{V_1 \cdot mL} \right)^2 + \left(\frac{C_2 \cdot dV_1^2}{(V_1^2) \cdot mL}\right)^2}}{V_1 \cdot mL} \right)^2 + \left(\frac{C_1 \cdot dV_1^2}{V_1^2 \cdot mL}\right)^2} \cdot V}{C^2} \right)^2} \quad \text{[Eq. 1]}$$

Supplementary equation 1. Formula employed for error calculation

Where: $m = mass;\ M = molar\ mass;\ C = 0.0017\ mM;\ V_1 = 10\ mL;\ V = 1\ mL$

Cl^-/Br^- poisoning experiments

1 mg (1.7 µmol, 1 eq) **1** and 12 mg (116.6 µmol, 73 eq) NaBr, respectively 7 mg (116.6 µmol, 73 eq) NaCl, are mixed in 1 mL DMSO:H_2O (5 v/v% H_2O) and stirred for 24h. Afterwards, 5 mL DMSO:H_2O (5 v/v% H_2O) are added, the solution transferred together with 700 mg (3.3 mmol) BMMI.OAc to a Parr-reactor. Then the reactor is flushed with CO_2 to remove residual air contamination, pressurized with 30 bar CO_2 and filled with H_2 to 60 bar. The reactor was heated to 80 °C using a heating mantle under hard stirring. After 4 h (72 h) the reactor was cooled down to room temperature and carefully vented. The reaction mixture was analysed directly by ^1H NMR spectroscopy in DMSO-d_6 to determine the amount of formic acid formed, using BMMI.OAc as an internal standard.

Initial screening method

1 mg (1.7 µmol, 1 eq) **1** is dissolved in 6 mL DMSO:H_2O (5 v/v% H_2O)/ 6 mL 1,4-dioxane:H_2O (5 v/v% H_2O). Then 700 mg (3.3 mmol) BMMI.OAc is added and the reaction mixture is transferred to a Parr-reactor. Then the reactor is flushed with CO_2 to remove residual air contamination, pressurized with 30 bar CO_2 and filled with H_2 to 60 bar. The reactor was heated to the desired temperature using a heating mantle under hard stirring. After 4 h (72 h) the reactor was cooled down to room temperature and carefully vented. The reaction mixture was analysed directly by ^1H NMR spectroscopy in DMSO-d_6 to determine the amount of formic acid formed, using BMMI.OAc as an internal standard.

General procedure for the hydrogenation of CO_2

From a stock solution containing the catalyst (0.17 mM, 0.017 mM or 0.0017 mM) 1 mL is added to 700 mg (3.3 mmol) BMMI.OAc and then loaded into a Parr reactor (50 mL). Then 5 mL of dioxane:water (5 v/v% water) from a stock solution was introduced into the reactor. The reactor was flushed with CO_2 gas in order to remove air. Afterwards, the reactor was filled with the desired amount of CO_2 and filled with H_2 gas, till the desired total pressure was reached at room temperature. The reactor was heated to the desired reaction temperature using a heating mantle under hard stirring. After the desired reaction time the reactor was cooled down to room temperature and carefully vented. The reaction mixture was analysed directly by ^{1}H NMR spectroscopy in DMSO-d_6 to determine the amount of formic acid formed, using BMMI.OAc as an internal standard.

Screening of the effect of water onto the hydrogenation of CO_2

From a stock solution containing the catalyst (0.017 mM) 1 mL is added to 700 mg (3.3 mmol) BMMI.OAc and then loaded into a Parr reactor (50 mL). Then 5 mL of dioxane:water with varying amounts of water from a stock solution was introduced into the reactor. The reactor was flushed with CO_2 gas in order to remove air. Afterwards, the reactor was filled with the desired amount of CO_2 and filled with H_2 gas, till the desired total pressure was reached at room temperature. The reaction was conducted at 120°C using a heating mantle under hard stirring. After the desired reaction time the reactor was cooled down to room temperature and carefully vented. The reaction mixture was analysed directly by ^{1}H NMR spectroscopy in DMSO-d_6 to determine the amount of formic acid formed, using BMMI.OAc as an internal standard.

Analyses for nanoparticle formation under reaction conditions

*Benzene test.*1 mg (1.7 µmol) of **1** is added to 700 mg (3.3 mmol) BMMI.OAc and then loaded into a Parr reactor (50 mL). Then 6 mL of dioxane:H_2O (5 v/v% H_2O) and 1 mL benzene with were introduced into the reactor. The reactor was flushed with H_2 gas in order to remove air. Afterwards, the reactor was filled with 50 bar H_2. The reaction was conducted at 120°C using a heating mantle under hard stirring. After 24h the reactor was cooled down to room temperature and carefully vented. The reaction mixture was analysed directly by ^{1}H NMR spectroscopy in DMSO-d$_6$ to determine the amount of formic acid formed, using BMMI.OAc as an internal standard.

DLS and TEM test. 1 mg (1.7 µmol) of **1** is added to 700 mg (3.3 mmol) BMMI.OAc and then loaded into a Parr reactor (50 mL). Then 6 mL of dioxane:H_2O (5 v/v% H_2O) was introduced into the reactor. The reactor was flushed with H_2 gas in order to remove air. Afterwards, the reactor was filled with 45 bar H_2 and 15 bar CO_2. The reaction was conducted at 140°C using a heating mantle under hard stirring. After 24h the reactor was cooled down to room temperature and carefully vented. The reaction mixture was analysed directly by ^{1}H NMR spectroscopy in DMSO-d$_6$ to determine the amount of formic acid formed, using BMMI.OAc as an internal standard. The reaction solution was screened for nanoparticles by Dynamic light scattering and TEM analysis. None of those experiments displayed nanoparticles under the investigated reaction conditions.

Screening of the effect of Sc(OTf)$_3$ on the hydrogenation of CO_2

From a stock solution containing the catalyst (0.017 mM) 1 mL is added to 700 mg (3.3 mmol) BMMI.OAc and then loaded into a Parr reactor (50 mL). Then 5 mL of dioxane:water (5 v/v% water) from a stock solution was introduced into the reactor. Then the desired amount of Sc(OTf)$_3$ was added into the reactor. The reactor was flushed with CO_2 gas in order to remove air. Afterwards, the reactor was filled with the desired amount of CO_2 and filled with H_2 gas, till the desired total pressure was reached at room temperature. The reaction was conducted at 120°C using a heating mantle under hard stirring. After the desired reaction time the reactor was cooled down to room temperature and carefully vented. The reaction mixture was analysed directly by ^{1}H NMR spectroscopy in DMSO-d_6 to determine the amount of formic acid formed, using BMMI.OAc as an internal standard.

Crystal structure determination of 1

A single crystal was selected and mounted using Fomblin® (YR-1800 perfluoropolyether oil) on a polymer-tipped MiTeGen MicroMountTM and cooled rapidly to 120 K in a stream of cold N_2 using an Oxford Cryosystems open flow cryostat.[8] Single crystal X-ray diffraction data were collected on an SuperNova Duo diffractometer (Atlas CCD area detector, mirror-monochromated Cu-Kα radiation source; λ = 1.54184 Å or mirror-monochromated Mo-Kα radiation source; λ = 0.71073 Å; ω scans). Cell parameters were refined from the observed positions of all strong reflections and absorption corrections were applied using a Gaussian numerical method with beam profile correction (CrysAlisPro).[9] The structure was solved within Olex2[10] by dual space iterative methods (SHELXT)[11] least squares refinement of the structure was carried using (SHELXL).[12] Structures were checked with checkCIF.[13] CCDC-1952963 contains the

supplementary data for these compounds. These data can be obtained free of charge from The Cambridge Crystallographic Data Centre via www.ccdc.cam.ac.uk/data_request/cif.

Crystal Data for $C_{24.5}H_{29}Br_{0.63}Cl_{1.29}N_5O_2Ru$ (M =622.38 g/mol): monoclinic, space group C2/m (no. 12), a = 8.9075(3) Å, b = 13.6660(4) Å, c = 22.4967(7) Å, β = 97.948(3)°, V = 2712.21(15) Å^3, Z = 4, T = 120(2) K, μ(CuKα) = 7.172 mm^{-1}, $Dcalc$ = 1.524 g/cm^3, 10836 reflections measured (7.936° ≤ 2Θ ≤ 149.108°), 2843 unique (R_{int}= 0.0387, R_{sigma} = 0.0241) which were used in all calculations. The final R_1 was 0.0338 (I > 2σ(I)) and wR_2 was 0.0841 (all data).

Refinement of single crystal structure

Both the coordinated and free bromide and chloride atoms were found to be substitutionally disordered. For each pair of atoms their occupancies were refined and constrained to sum to unity. Coordinated atoms Cl1 and Br1 were refined to values of 0.83(1) and 0.17(1) respectively. Coordinated atoms Cl2 and Br2 were refined to values of 0.62(1) and 0.38(1) respectively. Atoms Cl2 and Br2 were constrained to occupy the same position (EXYZ). Pairs of atoms Cl1/Br1 and Cl2/Br2 were constrained to have identical anisotropic displacement parameters (EADP) (Supplementary Figure 8).

The anisotropic displacement parameters of all carbon, nitrogen and oxygen atoms in the structure were treated with rigid bond restraints (RIGU).

The toluene solvent molecule is disordered by symmetry over four orientations, each necessarily refined with a fixed occupancy of 0.25. Geometric restraints have been applied to the molecule reflecting its mirror plane symmetry and planarity (SADI, FLAT). The anisotropic displacement parameters have been restrained to be similar (SIMU) and more isotropic in character (ISOR).

We observe that **1** crystallises with toluene. Structurally, the NHC **1** wingtips display identical bond lengths (2.075 Å), and the bonds in the NHC moiety are identical. Furthermore, **1** displays a symmetrical plane along the N11-Ru-C1A-O2A axis. The N11-Ru bond lengths is 2.011 Å long, which is identical to previously reported Ru-N(pyr) bond lengths in CNC-pincer complexes.[14] A significant trans-effect can be observed, i.e. the Ru-C1A (1.895 Å) bond is significantly shorter as the corresponding Ru-C1B (1.937 Å) bond lengths and the C1B-O1B (1.087 Å) bond is significantly shorter than the C1A-O1A (1.110 Å) bond length. Note that the Ru-C1A bond length is identical to Ru-CO bond lengths in similar Ru-CNC pincer complexes.[14] A Ru-Br1 bond length of 2.636 Å is found whilst the Ru-Cl1 bond length is comparatively compressed to 2.410 Å. Most bond angles display almost identical angles (90°). Exceptions are the NHC wingtips. Here compression of the C21-Ru-N11 (76°) bond and consequently stretching of the C21-Ru-C1A (103°) can be found. In general, the structural arrangements are similar as already described for other complexes.[14] All the data is summarised in Supplementary Tables 5-11.

Supplementary note 1 Comparison of initial screening with thermodynamic properties of the system

The thermodynamic properties for the catalytic hydrogenation of CO_2 to formic acid have been determined previously.[7] Using the data published in reference 7 the concentrations of FA observed with 1 can be compared to the theoretical maximum of FA achievable in the solvent IL system, see figure below. Note that the thermodynamic equilibrium is determined for a system at $P(CO_2) = P(H_2) = 30$ bar in DMSO:H_2O (5 v/v% H_2O) with BMMI.OAc (0.6M) dissolved in the solvent system. Accordingly, small deviations with the present system can be expected. The concentration can be calculated according to the equation 1, whereas the concentration of CO_2 and H_2 can be calculated with the henry equation using data provided in reference 7. The results have been plotted in Supplementary Figure 6

$$[FA] = [CO_2][H_2]\exp(-\frac{\Delta H}{RT} + \frac{\Delta S}{R}) \qquad \text{[Eq. 2]}$$

Supplementary Equation 2. Formula employed to calculate concentration of formic acid under equilibrium as a function of temperature.

Supplementary note 2

As highlighted in the main manuscript, the rate determining step can be assumed to be related to the CO_2 insertion into the formed metal hydride bond. In previous studies it has been demonstrated that CO_2 can insert into a metal bond via an inner or outer sphere mechanism. The outer sphere mechanism can be further facilitated by utilizing Lewis acids which stabilise the charged intermediate/transition state present in the outer sphere mechanism. Supplementary Figure 7 suggests the mechanism proposed.

Supplementary References

1 Moret, S., Dyson, P. J. & Laurenczy, G. Direct synthesis of formic acid from carbon dioxide by hydrogenation in acidic media. *Nat. Commun.* **5**, 4017 (2014).

2 Rohmann, K. *et al.* Hydrogenation of CO2 to Formic Acid with a Highly Active Ruthenium Acriphos Complex in DMSO and DMSO/Water. *Angew. Chem. Int. Ed.* **55**, 8966-8969 (2016).

3 Hayashi, H., Ogo, S. & Fukuzumi, S. Aqueous hydrogenation of carbon dioxide catalysed by water-soluble ruthenium aqua complexes under acidic conditions. *Chem. Commun.*, 2714-2715 (2004).

4 Ogo, S., Kabe, R., Hayashi, H., Harada, R. & Fukuzumi, S. Mechanistic investigation of CO2 hydrogenation by Ru(ii) and Ir(iii) aqua complexes under acidic conditions: two catalytic systems differing in the nature of the rate determining step. *Dalton Trans.*, 4657-4663 (2006).

5 Zhao, G. & Joó, F. Free formic acid by hydrogenation of carbon dioxide in sodium formate solutions. *Catal. Commun.* **14**, 74-76 (2011).

6 Wesselbaum, S., Hintermair, U. & Leitner, W. Continuous-Flow Hydrogenation of Carbon Dioxide to Pure Formic Acid using an Integrated scCO2 Process with Immobilized Catalyst and Base. *Angew. Chem.* **124**, 8713-8716 (2012).

7 Weilhard, A., Qadir, M. I., Sans, V. & Dupont, J. Selective CO2 Hydrogenation to Formic Acid with Multifunctional Ionic Liquids. *ACS Catal.* **8**, 1628-1634 (2018).

8 Cosier, J. & Glazer, A. M. *J. Appl. Crystallogr.* **19**, 105−107 (1986).

9 Rigaku Oxford Diffraction, C. S. s., version 1.171.40.45a, Rigaku Corporation, Oxford, UK., (2018).

10 Dolomanov, O. V., Bourhis, L. J., Gildea, R. J, Howard, J. A. K. & Puschmann, H. *J. Appl. Crystallogr.* **42**, 339-341 (2009).

11 Sheldrick, G. M. *Acta Cryst.* **A71**, 3-8 (2015).

12 Sheldrick, G. M. *Acta Cryst.* **C71**, 3-8 (2015).

13 *http://checkcif.iucr.org*.

14 Poyatos, M., Mata, J. A., Falomir, E., Crabtree, R. H. & Peris, E. New Ruthenium(II) CNC-Pincer Bis(carbene) Complexes: Synthesis and Catalytic Activity. *Organometallics* **22**, 1110-1114 (2003).
