# Supplementary material for: Efficient carbon dioxide hydrogenation to formic acid with buffering ionic liquids
Source: Nat Commun. 2021 Jan 11;12:231. doi: 10.1038/s41467-020-20291-0 (PMC7801478; doi:10.1038/s41467-020-20291-0)

checkCIF/PLATON report

Structure factors have been supplied for datablock(s) ruaswb

THIS REPORT IS FOR GUIDANCE ONLY. IF USED AS PART OF A REVIEW PROCEDURE FOR PUBLICATION, IT SHOULD NOT REPLACE THE EXPERTISE OF AN EXPERIENCED CRYSTALLOGRAPHIC REFEREE.

No syntax errors found.   CIF dictionary   Interpreting this report

Datablock: ruaswb

```
Bond precision:  C-C = 0.0038 A          Wavelength=1.54184

Cell:            a=8.9075(3)     b=13.6660(4)      c=22.4967(7)
                 alpha=90        beta=97.948(3)    gamma=90
Temperature:     120 K
```

	Calculated	Reported
Volume	2712.22(15)	2712.21(15)
Space group	C 2/m	C 1 2/m 1
Hall group	-C 2y	-C 2y
Moiety formula	2(C21 H25 Br0.17 Cl0.83 N5 O2 Ru), C7 H8, 0.752(Br), 1.248(Cl)	C21 H25 Br0.17 Cl0.83 N5 O2 Ru, 0.38(Br), 0.62(Cl), 0.5(C7 H8)
Sum formula	C49 H58 Br1.09 Cl2.91 N10 O4 Ru2	C24.50 H29 Br0.63 Cl1.29 N5 O2 Ru
Mr	1243.53	622.38
Dx,g cm-3	1.523	1.524
Z	2	4
Mu (mm-1)	7.184	7.172
F000	1259.3	1259.0
F000’	1262.92	
h,k,lmax	11,17,28	10,16,27
Nref	2888	2843
Tmin,Tmax	0.235,0.601	0.148,0.890
Tmin’	0.046	

```
Correction method= # Reported T Limits: Tmin=0.148 Tmax=0.890
AbsCorr = GAUSSIAN

Data completeness= 0.984          Theta(max)= 74.554

R(reflections)= 0.0338( 2830)     wR2(reflections)= 0.0841( 2843)

S = 1.097                   Npar= 226
```

The following ALERTS were generated. Each ALERT has the format
test-name_ALERT_alert-type_alert-level.
Click on the hyperlinks for more details of the test.

Alert level C

```
PLAT041_ALERT_1_C Calc. and Reported SumFormula    Strings  Differ     Please Check
PLAT077_ALERT_4_C Unitcell Contains Non-integer Number of Atoms ..     Please Check
PLAT250_ALERT_2_C Large U3/U1 Ratio for Average U(i,j) Tensor ....        3.7 Note
PLAT331_ALERT_2_C Small Aver Phenyl C-C Dist C1S       -C6S      .       1.37 Ang.
PLAT911_ALERT_3_C Missing FCF Refl Between Thmin & STh/L=    0.600          3 Report
PLAT971_ALERT_2_C Check Calcd Resid. Dens.  0.79A   From Ru1             1.67 eA-3
```

Alert level G

```
FORMU01_ALERT_1_G  There is a discrepancy between the atom counts in the
            _chemical_formula_sum and _chemical_formula_moiety. This is
            usually due to the moiety formula being in the wrong format.
            Atom count from _chemical_formula_sum:   C24.5 H29 Br0.63 Cl1.29 N5 O2
            Atom count from _chemical_formula_moiety:C24.5 H29 Br0.55 Cl1.45 N5 O2
FORMU01_ALERT_2_G  There is a discrepancy between the atom counts in the
            _chemical_formula_sum and the formula from the _atom_site* data.
            Atom count from _chemical_formula_sum:C24.5 H29 Br0.63 Cl1.29 N5 O2 Ru
            Atom count from the _atom_site data:  C24.5 H29 Br0.546 Cl1.454 N5 O2
CELLZ01_ALERT_1_G Difference between formula and atom_site contents detected.
CELLZ01_ALERT_1_G ALERT: Large difference may be due to a
            symmetry error - see SYMMG tests
           From the CIF: _cell_formula_units_Z    4
           From the CIF: _chemical_formula_sum  C24.50 H29 Br0.63 Cl1.29 N5 O2 Ru
           TEST: Compare cell contents of formula and atom_site data

           atom    Z*formula  cif sites diff
           C         98.00     98.00    0.00
           H        116.00    116.00    0.00
           Br         2.52      2.18    0.34
           Cl         5.16      5.82   -0.66
           N         20.00     20.00    0.00
           O          8.00      8.00    0.00
           Ru         4.00      4.00    0.00
PLAT002_ALERT_2_G Number of Distance or Angle Restraints on AtSite          7 Note
PLAT003_ALERT_2_G Number of Uiso or Uij Restrained non-H Atoms ...         24 Report
PLAT042_ALERT_1_G Calc. and Reported MoietyFormula Strings  Differ     Please Check
PLAT045_ALERT_1_G Calculated and Reported Z Differ by a Factor ...       0.50 Check
PLAT068_ALERT_1_G Reported F000 Differs from Calcd (or Missing)...     Please Check
PLAT083_ALERT_2_G SHELXL Second Parameter in WGHT  Unusually Large      11.17 Why ?
PLAT171_ALERT_4_G The CIF-Embedded .res File Contains EADP Records          2 Report
PLAT174_ALERT_4_G The CIF-Embedded .res File Contains FLAT Records          1 Report
PLAT176_ALERT_4_G The CIF-Embedded .res File Contains SADI Records          4 Report
PLAT178_ALERT_4_G The CIF-Embedded .res File Contains SIMU Records          2 Report
PLAT186_ALERT_4_G The CIF-Embedded .res File Contains ISOR Records          1 Report
PLAT187_ALERT_4_G The CIF-Embedded .res File Contains RIGU Records          2 Report
PLAT232_ALERT_2_G Hirshfeld Test Diff (M-X)  Ru1      --Br1      .        5.8 s.u.
PLAT232_ALERT_2_G Hirshfeld Test Diff (M-X)  Ru1      --Cl1      .        5.9 s.u.
PLAT232_ALERT_2_G Hirshfeld Test Diff (M-X)  Ru1      --C1A      .        9.0 s.u.
PLAT300_ALERT_4_G Atom Site Occupancy of C1S        Constrained at       0.25 Check
PLAT300_ALERT_4_G Atom Site Occupancy of C2S        Constrained at       0.25 Check
PLAT300_ALERT_4_G Atom Site Occupancy of C3S        Constrained at       0.25 Check
PLAT300_ALERT_4_G Atom Site Occupancy of C4S        Constrained at       0.25 Check
PLAT300_ALERT_4_G Atom Site Occupancy of C5S        Constrained at       0.25 Check
PLAT300_ALERT_4_G Atom Site Occupancy of C6S        Constrained at       0.25 Check
```

```
PLAT300_ALERT_4_G Atom Site Occupancy of C7S        Constrained at       0.25 Check
PLAT300_ALERT_4_G Atom Site Occupancy of H7SA       Constrained at       0.25 Check
PLAT300_ALERT_4_G Atom Site Occupancy of H7SB       Constrained at       0.25 Check
PLAT300_ALERT_4_G Atom Site Occupancy of H2S        Constrained at       0.25 Check
PLAT300_ALERT_4_G Atom Site Occupancy of H7SC       Constrained at       0.25 Check
PLAT300_ALERT_4_G Atom Site Occupancy of H3S        Constrained at       0.25 Check
PLAT300_ALERT_4_G Atom Site Occupancy of H4S        Constrained at       0.25 Check
PLAT300_ALERT_4_G Atom Site Occupancy of H5S        Constrained at       0.25 Check
PLAT300_ALERT_4_G Atom Site Occupancy of H6S        Constrained at       0.25 Check
PLAT301_ALERT_3_G Main Residue  Disorder ..............(Resd  1  )         6% Note
PLAT302_ALERT_4_G Anion/Solvent/Minor-Residue Disorder (Resd  2  )       100% Note
PLAT302_ALERT_4_G Anion/Solvent/Minor-Residue Disorder (Resd  3  )       100% Note
PLAT302_ALERT_4_G Anion/Solvent/Minor-Residue Disorder (Resd  4  )       100% Note
PLAT304_ALERT_4_G Non-Integer Number of Atoms in ...... Resd  2          3.75 Check
PLAT304_ALERT_4_G Non-Integer Number of Atoms in ...... Resd  3          0.19 Check
PLAT304_ALERT_4_G Non-Integer Number of Atoms in ...... Resd  4          0.31 Check
PLAT720_ALERT_4_G Number of Unusual/Non-Standard Labels ..........          3 Note
PLAT789_ALERT_4_G Atoms with Negative _atom_site_disorder_group  #         15 Check
PLAT802_ALERT_4_G CIF Input Record(s) with more than 80 Characters          3 Info
PLAT860_ALERT_3_G Number of Least-Squares Restraints .............        236 Note
PLAT910_ALERT_3_G Missing # of FCF Reflection(s) Below Theta(Min).          1 Note
PLAT912_ALERT_4_G Missing # of FCF Reflections Above STh/L=  0.600         29 Note
PLAT933_ALERT_2_G Number of OMIT Records in Embedded .res File ...          3 Note
PLAT961_ALERT_5_G Dataset Contains no Negative Intensities .......     Please Check
PLAT978_ALERT_2_G Number C-C Bonds with Positive Residual Density.          2 Info
```

```
   0 ALERT level A = Most likely a serious problem - resolve or explain
   0 ALERT level B = A potentially serious problem, consider carefully
   6 ALERT level C = Check. Ensure it is not caused by an omission or oversight
  50 ALERT level G = General information/check it is not something unexpected

   7 ALERT type 1 CIF construction/syntax error, inconsistent or missing data
  12 ALERT type 2 Indicator that the structure model may be wrong or deficient
   4 ALERT type 3 Indicator that the structure quality may be low
  32 ALERT type 4 Improvement, methodology, query or suggestion
   1 ALERT type 5 Informative message, check
```

It is advisable to attempt to resolve as many as possible of the alerts in all categories. Often the minor alerts point to easily fixed oversights, errors and omissions in your CIF or refinement strategy, so attention to these fine details can be worthwhile. In order to resolve some of the more serious problems it may be necessary to carry out additional measurements or structure refinements. However, the purpose of your study may justify the reported deviations and the more serious of these should normally be commented upon in the discussion or experimental section of a paper or in the "special_details" fields of the CIF. checkCIF was carefully designed to identify outliers and unusual parameters, but every test has its limitations and alerts that are not important in a particular case may appear. Conversely, the absence of alerts does not guarantee there are no aspects of the results needing attention. It is up to the individual to critically assess their own results and, if necessary, seek expert advice.

Publication of your CIF in IUCr journals

A basic structural check has been run on your CIF. These basic checks will be run on all CIFs submitted for publication in IUCr journals (*Acta Crystallographica*, *Journal of Applied Crystallography*, *Journal of Synchrotron Radiation*); however, if you intend to submit to *Acta Crystallographica Section C* or *E* or *IUCrData*, you should make sure that full publication checks are run on the final version of your CIF prior to submission.

Publication of your CIF in other journals

Please refer to the *Notes for Authors* of the relevant journal for any special instructions relating to CIF submission.

PLATON version of 17/03/2019; check.def file version of 04/03/2019

Datablock ruaswb - ellipsoid plot

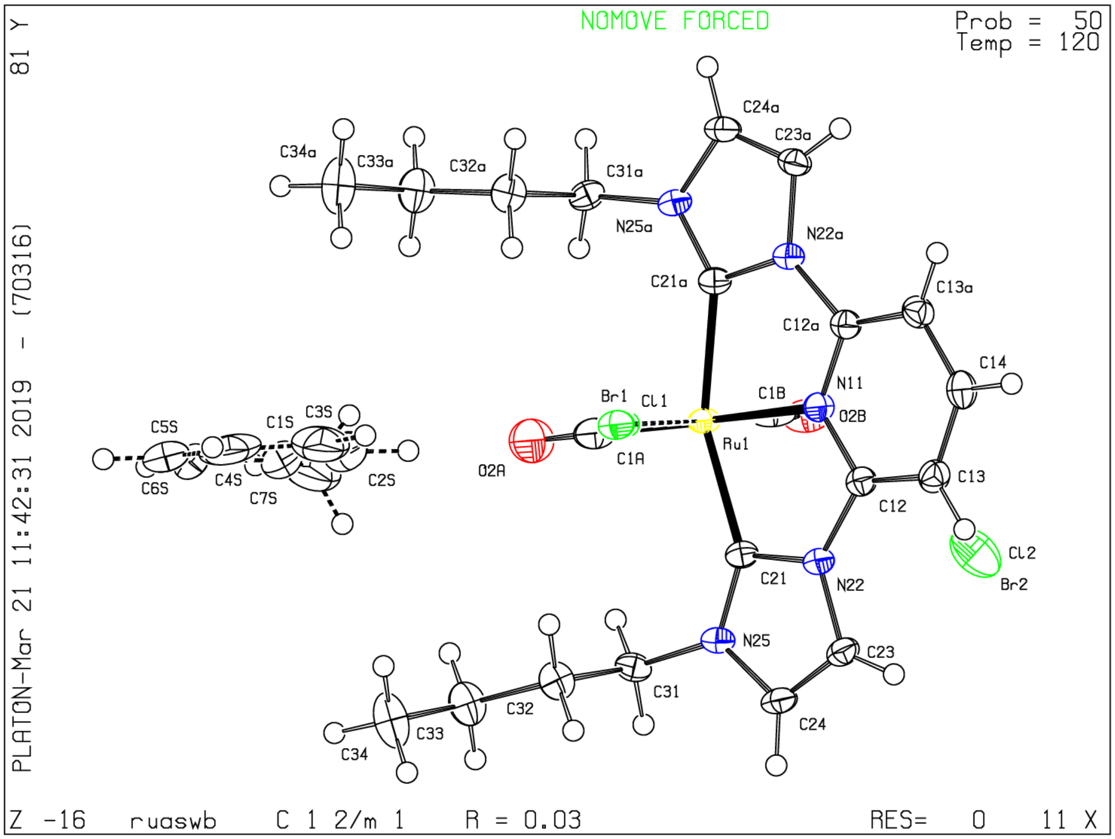

Supplement: Supplementary file 2 — Source Data [file 41467_2020_20291_MOESM2_ESM.zip › Crystal structure refinement information 1.pdf]
